# Karrikin Improves Osmotic and Salt Stress Tolerance *via* the Regulation of the Redox Homeostasis in the Oil Plant *Sapium sebiferum*

**DOI:** 10.3389/fpls.2020.00216

**Published:** 2020-03-24

**Authors:** Faheem Afzal Shah, Xiao Wei, Qiaojian Wang, Wenbo Liu, Dongdong Wang, Yuanyuan Yao, Hao Hu, Xue Chen, Shengwei Huang, Jinyan Hou, Ruiju Lu, Chenghong Liu, Jun Ni, Lifang Wu

**Affiliations:** ^1^Key Laboratory of the High Magnetic Field and Ion Beam Physical Biology, Hefei Institutes of Physical Science, Chinese Academy of Sciences, Hefei, China; ^2^Biotechnology Research Institute, Shanghai Academy of Agricultural Sciences, Shanghai, China; ^3^Taihe Experimental Station, Hefei Institutes of Physical Science, Chinese Academy of Sciences, Taihe, China

**Keywords:** karrikins, seed germination, salt stress, osmotic stress, redox homeostasis, abscisic acid signaling

## Abstract

Karrikins are reported to stimulate seed germination, regulate seedling growth, and increase the seedling vigor in abiotic stress conditions in plants. Nevertheless, how karrikins alleviate abiotic stress remains largely elusive. In this study, we found that karrikin (KAR^1^) could significantly alleviate both drought and salt stress in the important oil plant *Sapium sebiferum*. KAR^1^ supplementation in growth medium at a nanomolar (nM) concentration was enough to recover seed germination under salt and osmotic stress conditions. One nanomolar of KAR^1^ improved seedling biomass, increased the taproot length, and increased the number of lateral roots under abiotic stresses, suggesting that KAR^1^ is a potent alleviator of abiotic stresses in plants. Under abiotic stresses, KAR^1^-treated seedlings had a higher activity of the key antioxidative enzymes, such as superoxide dismutase, peroxidase, catalase, and ascorbate peroxidase, in comparison with the control, which leads to a lower level of hydrogen peroxide, malondialdehyde, and electrolyte leakage. Moreover, the metabolome analysis showed that KAR^1^ treatment significantly increased the level of organic acids and amino acids, which played important roles in redox homeostasis under stresses, suggesting that karrikins might alleviate abiotic stresses *via* the regulation of redox homeostasis. Under abiotic stresses, applications of karrikins did not increase the endogenous abscisic acid level but altered the expression of several ABA signaling genes, such as *SNF1-RELATED PROTEIN KINASE2.3*, *SNF1-RELATED PROTEIN KINASE2.6*, *ABI3*, and *ABI5*, suggesting potential interactions between karrikins and ABA signaling in the stress responses. Conclusively, we not only provided the physiological and molecular evidence to clarify the mechanism of karrikins in the regulation of stress adaptation in *S. sebiferum* but also showed the potential value of karrikins in agricultural practices, which will lay a foundation for further studies about the role of karrikins in abiotic stress alleviation in plants.

## Introduction

Abiotic stresses, such as drought and salt, are the major constraints to crop productivity worldwide. The stresses could cause a wide range of morphological, physiological, and anatomical disorders in plants (Foley et al., 2011). Though plants have established a coping mechanism to certain abiotic stresses, this process is energy-consuming, resulting in stunted plant growth and low yield. Abiotic stresses could cause crop yield loss by 50–80%, depending on the crop and the geographical location (Shinozaki et al., 2015). Thus, to ensure world food security, it is emergent to develop stress-tolerant crops or to find ways to strengthen the crops and stabilize the crop productivity under stresses (Zhang et al., 2018). The identification of stress regulatory genes and compounds involved in the stress signaling network will be crucial for the improvement of crop productivity.

To maintain growth under abiotic stresses, plants often depend on phytohormones such as abscisic acid (ABA), which plays a prominent role in the alleviation of abiotic stresses and controls the downstream stress responses (Jian Kang, 2016). Under abiotic stresses, such as osmotic and salt stresses, endogenous ABA levels are increased, showing that drought and salt stresses are the most important environmental signals upregulating the transcription of the ABA biosynthesis genes (Li Ming and Jian Kang, 2003). Meanwhile, ABA biosynthesis genes such as *NCED3* or *NCED6* showed a regulatory role in osmotic and salt stresses (Qin and Zeevaart, 1999; Thompson et al., 2010; Huang et al., 2018). The class III *SNF-1-related protein kinases 2* (*SnRK2s*) are involved in ABA signaling and discovered as responsive to abiotic stresses (Kulik et al., 2011). Furthermore, it has also been reported that ABA signaling genes such as *ABA Insensitive 3* (*ABI3*) and *ABA Insensitive 5* (*ABI5*) function together to alleviate the tolerance under abiotic stresses (Nakamura et al., 2001; Skubacz et al., 2016).

Karrikins are a family of closely related chemical compounds that are present in charred or burnt plant material and its smoke. Pyrolysis of cellulose and simple sugars also produce karrikins (Flematti et al., 2011). To date, in smoke, six karrikins have been discovered and named as KAR^1^, KAR^2^, KAR^3^, KAR^4^, KAR^5^, and KAR^6^. Among all karrikins, KAR^1–^KAR^4^ have been considered as the most active forms of karrikins (Nelson et al., 2012). Karrikins promote seed germination and photomorphogenesis, while they negatively regulate hypocotyl elongation in many plants (Nelson et al., 2010, 2012; Waters et al., 2017). Under red light, karrikins inhibit the hypocotyl length in a dose-dependent manner; the hypocotyl length of *Arabidopsis* seedlings treated with 1 μM KAR^2^ was almost half that of the hypocotyl length of the untreated seedlings (Nelson et al., 2010; Waters and Smith, 2013). The regulation of cotyledon expansion and chlorophyll accumulation by karrikins was also detected in the seedlings of *Brassica tournefourtii* and *Lactuca sativa* (Nelson et al., 2010).

Recently, karrikins have also been discovered to play a role against abiotic stresses. Karrikins played an essential role in both the early chilling and the chilling response of tea plants (Zhao et al., 2012). In tomato, seeds primed with butenolide (karrikin) produced significantly (*P* ≤ 0.05) more vigorous seedlings than the water-primed seeds. The vigor indices of seedlings having been primed with butenolide were significantly higher under various stress conditions (salt, osmoticum, or temperature) (Jain and Van, 2007). *KARRIKINS INSENSITIVE2* (*KAI2*), which encodes an α/β-fold hydrolase, has been discovered as a receptor gene for karrikins (Scaffidi et al., 2012; Zhao et al., 2013). KAI2 has a hydrophobic pocket that contains a conserved catalytic triad (Kagiyama et al., 2013) and also hydrolytic pocket which binds to the karrikins (Boyer et al., 2012; Hamiaux et al., 2012). *KAI2* gene was demonstrated to be involved in the regulation of cuticle formation, stomatal closure, anthocyanin biosynthesis, and membrane integrity, which contributes to plant adaptation to the drought stress (Li et al., 2017). Recently, it has been found that the karrikin-*KAI2* signaling system protected *Arabidopsis* against abiotic stress by providing stress tolerance and by inhibiting germination under unfavorable conditions (Wang et al., 2018).

*Sapium sebiferum* L., which belongs to the Euphorbiaceae family, is native to eastern Asia (Esser, 2002). Its fruits produce a highly saturated fatty acid in the tallow layer and highly unsaturated oil in the seed (Boldor et al., 2010). It has been estimated that *S. sebiferum* could produce 4,700 L of oil per hectare every year (Webster et al., 2006; Boldor et al., 2010). Due to its high seed yield, *S. sebiferum* has gotten attention as a source for biodiesel (Gao et al., 2016). Soil salinity and osmotic stress could reduce the seed germination and seedling growth of various crops, including oil crops such as sunflower and soybean (Luan et al., 2014; Shu et al., 2017). Under high salinity, reactive oxygen species (ROS) accumulates in the seed, causing oxidative damage and delaying germination, while antioxidants can reduce the concentration of ROS and eliminate the inhibitory effect of ROS (Lee et al., 2010). Previous studies demonstrated that karrikin stimulated the activities of antioxidant enzymes, such as superoxide dismutase (SOD) and catalase (CAT), during the seed germination process in *Avena fatua* caryopses (Cembrowska-Lech et al., 2015). The importance of the antioxidant enzymes in the maintenance of ROS homeostasis is well established in any kind of combination of abiotic stresses (Ni et al., 2018b). We hypothesized that KAR^1^ could directly regulate the core ROS-scavenging cycle by increasing the activity of the antioxidant enzymes under abiotic stresses.

In this study, we used KAR^1^ to validate our hypothesis and found that osmotic and salt stresses inhibited seed germination and seedling growth in *S. sebiferum*, which were recovered by KAR^1^ supplementation in the growth medium. We measured plant mortality rate, shoot length, fresh weight, photochemical efficiency of the PSII, root fresh weight, root length, lateral root/plant, and lateral root length in *S. sebiferum* under salt and osmotic stresses. We investigated the ROS, malondialdehyde (MDA), and antioxidant enzyme contents under salt and osmotic stresses, which supported our hypothesis. We further investigated the KAR^1^-regulated metabolome and the expression pattern of karrikins, ABA, and stresses-related genes in the seedlings under stresses. This study provided evidences of the physiological and the molecular mechanisms of karrikins in the regulation of stress adaptation in *S. sebiferum* and also showed the high potential value of karrikins in agriculture.

## Materials and Methods

### Seed Material and Seedling Preparations and Growing Conditions

*Sapium sebiferum* seeds were harvested from plants grown in the experimental farm of the Hefei Institute of Physics, Chinese Academy of Sciences, Anhui, China. Before use, the seeds were stored in nylon bags at room temperature. White tallow was removed by washing the seeds with 1% sodium hydroxide (NaOH, CAS# 1310-73-2, Shanghai Chemical Reagent Co., Ltd.). Sulfuric acid scarification was done by dipping the seeds in concentrated (98.99%) sulfuric acid (CAS 7664-93-9, Sinopharm Chemical Reagent Co., Ltd.) at 4°C for 30 min. After the sulfuric acid treatment, the seeds were washed five times in running tap water by manual shaking. The sulfuric acid-scarified seeds were primed in distilled water and placed at 22°C overnight. The primed seeds were sown in 10 × 10-cm pots that have peat and perlite (1:1) medium. The germination condition was maintained as follows: 25°C, 16 h light/8 h dark, 150 μmol m^–2^ s^–1^ light strength, as previously described (Shah et al., 2018).

### KAR^1^ Stock and Working Solution Preparation

KAR^1^ (3-methyl 2H-furo [2,3-c] pyran-2-one) was purchased from Toronto Research Chemicals Canadan/WuXi AppTec. The stock solution (10 mM) was prepared by dissolving the KAR^1^ in absolute methanol (CAS# 67-56-1, Titan Scientific Co., Ltd. Shanghai, China). Then, 1 ml of stock solution was added into 10 ml of distilled water to make the sub-stock solution, and this solution was used for preparing the desired concentrations in MS medium or Hoagland nutrient solution (Qingdao Hope Bio-Technology Co., Ltd. Shandong, China). The stock and sub-stock solutions were stored at −20°C.

### Salt and Osmotic Stress Application in Seed and Seedlings

For the germination investigation under stresses, the seeds were de-coated with sharp scissors and sterilized by washing twice with 70% ethyl alcohol (CAS# 64-17-5, Titan Scientific Co., Ltd. Shanghai, China) for 30 s followed by incubation in 20% sodium hypochlorite [NaClO, CAS# 7681-52-9, Sangon Biotech (Shanghai) Co., Ltd.] for 10 min. After that, the seeds were washed three times with sterilized water and dried by blotting in sterilized filter papers. For the seed germination experiment, half of the MS medium (pH = 7) was supplemented with 0, 100, 150, and 200 mM NaCl for salt stress and 0, 100, 200, and 300 mM mannitol for osmotic stress. In a preliminary experiment, we found that supplementation of 1 nM KAR^1^ in the seed germination medium significantly improved seed germination in *S. sebiferum* (Supplementary Data 1). Therefore, 1 nM KAR^1^ was supplemented in the half MS medium in 8 × 8-cm Petri plates containing NaCl or mannitol. The seed germination test was carried out in five replicates (Petri plates, 12 seeds in each plate). Protrusion of radicle from the micropyle was considered as the criterion of seed germination. Germination data were recorded 10 days after imbibition. All seed germination pictures were taken with a digital camera, NIKON D90 with NIKON DX AF-S NIKKOR 18–105 mm lens (Shah et al., 2018).

For seedling growth, 5-day-old seedlings were transplanted to 15 × 10 × 6-cm pots containing 800 ml of 1/5 Hoagland nutrient solution with or without 1 nM KAR^1^. After 7 days of transplantation, the seedlings were used for stress treatment by completely replacing the growth medium with the newly prepared medium containing salt or mannitol, with or without KAR^1^. The medium was renewed every day. Pictures and growth parameters data were taken 15 days after the treatments. The lengths of the shoot and the root were measured with a ruler, and lateral root length was measured with ImageJ 1.52a.

### ABA Content Determination

ABA content was determined following the previously reported method (Tang et al., 2011). Briefly, 1 g of fresh sample was ground in liquid nitrogen, homogenized, and then extracted overnight with 30 ml of 80% cold aqueous methanol in darkness at 4°C. The extract was centrifuged at 5,000 rpm at 4°C for 15 min, and the supernatant was collected. The remnant was extracted three times. After that, the total methanolic extract was dried in a stream of nitrogen gas and then dissolved in 2 ml of methanol. ABA was measured by the injection of the extract into a reverse-phase high-performance liquid chromatography (HPLC), with a methanol gradient in 0.6% acetic acid. The parameters for the HPLC were set as previously described by Tang et al. (2011).

### Metabolomics Analysis

Metabolomics analysis was carried out following the method by Tobias et al. (2009). In detail, three biological replicates from three different seedlings were collected for each treatment, a 50-mg sample was transferred into a 2-ml tube, followed by adding 450 μl of pre-cooled extraction mixture [methanol/dH_2_O (v:v) = 3:1] with 10 μl internal standard (adonitol, 0.5 mg/ml stock). The samples were vortexed for 30 s and homogenized with a ball mill for 4 min at 35 Hz, followed by ultrasonication for 5 min in ice water. After centrifugation at 4°C for 15 min at 10,000 rpm, 300 μl of supernatant was transferred to a fresh tube. To prepare the quality control (QC) sample, 50 μl of each sample was taken out and combined. After evaporation in a vacuum concentrator, 80 μl of methoxyamination hydrochloride (20 mg/ml in pyridine) was added and then incubated at 80°C for 30 min, then derivatized by 100 μl of *N*, *O*-bis (trimethylsilyl)-trifluoroacetamide BSTFA reagent (1% trimethylchlorosilane, v/v) at 70°C for 1.5 h. After gradually cooling to room temperature, 5 μl of fatty acid methyl esters (in chloroform) was added to the QC sample. All of the samples were then analyzed by gas chromatography coupled with time-of-flight mass spectrometry.

### Metabolomics Data Processing

Raw data analysis, including peak extraction, baseline adjustment, deconvolution, alignment, and integration, was finished using ChromaTOF (V 4.3x, LECO) software, and LECO-Fiehn Rtx5 database was used for metabolite identification by matching the mass spectrum and retention index. Finally, the peaks detected in less than half of the QC samples or RSD >30% in the QC samples were removed (Dunn et al., 2011). In the following, the missing value was filled using a small value that was half of the minimum positive value in the original data. Then, the data were filtered by interquantile range, and the total mass of the signal integration area was normalized for each sample. Differential metabolites were found using one-way ANOVA (*P* < 0.05) followed by *post hoc* Tukey’s HSD test. Subsequently, heatmap and Venn diagram were constructed on the bases of the changes in metabolite concentrations in each treatment compared with the control (Khan et al., 2019). All metabolomics data were normalized and analyzed by using the online website MetaboAnalyst^1^.

### Biochemical Analysis and Photochemical Efficiency of the PSII Measurement

For biochemical analysis, the root samples were taken from the seedlings after 3, 6, and 9 days under salt and osmotic stress treatment (150 mM NaCl and 200 mM mannitol, respectively). The MDA, hydrogen peroxide (H_2_O_2_), SOD, peroxidase (POD), CAT, and ascorbate peroxidase (APX) contents were determined by using an MDA assay kit, a plant soluble sugar content test kit, an H_2_O_2_ assay kit, an SOD assay kit, a POD assay kit, a CAT assay kit, and an APX assay kit (Nanjing Jiancheng Bioengineering Institute, Nanjing, China), following a protocol provided with a specific kit, as previously described by Ni et al. (2018b). The maximum photochemical efficiency of PSII (Fv/Fm) was calculated according to a previous method (Yin et al., 2018).

### Histological Staining of H_2_O_2_ and Electrolyte Leakage Determination

3,3′-Diaminobenzidine (DAB) staining method was used to detect *in situ* hydrogen peroxide as previously described by Hasan et al. (2015). The detached *S. sebiferum* leaves of the 20-day-old plant were immersed overnight in DAB (1 mg/ml, pH 3.8) solution. After incubating overnight at room temperature, the *S. sebiferum* leaves were then submerged in absolute ethanol for 12 h to wash off the chlorophyll.

Electrolyte leakage of seedlings was measured after growing the seedlings for 12 days under salt and osmotic stresses according to the method by Nishiyama et al. (2011). In detail, the aerial parts of five seedlings for each treatment (12 days after the treatments) were taken, the plant samples were then moved to the 50-ml tubes containing 40 ml of double-distilled water for 24 h. The electrical conductivity (EC) of water was measured with an EC meter. Then, the tube having 40 ml of water was autoclaved for 20 min at 121°C, and the electrical conductivity was measured again. The following equation was used to measure the electrolyte leakage percentage:

$$Electrolyteleakage\left(\%\right)=\frac{\mathrm{Electrical}\,\mathrm{conductivity}\,\mathrm{before}\,\mathrm{autoclave}}{\mathrm{Electrical}\,\mathrm{conductivity}\,\mathrm{after}\,\mathrm{autoclave}}\times 100.$$

### RNA Extraction and Quantitative Real-Time PCR

The full sequences of *S. sebiferum* genes were identified by local blast using *Arabidopsis* amino acid sequences as a reference in blast-2.2.31. A local blast library was constructed by using *S. sebiferum* flower–bud transcriptome (GenBank Accession ID: SRX656554) (Yang et al., 2015; Ni et al., 2018a). The lists of all genes’ full mRNA sequences are available in Supplementary Data 1. The quantitative PCR primers were designed by using primer premier 6, and a list of all the primers is provided in Supplementary Table 1. Fifteen-day-old seedlings, growing in 1/5 Hoagland nutrient solution, were subjected to osmotic stress (mimicked by 200 mM mannitol) and salt stress (mimicked by 200 mM NaCl). Samples with three biological replicates were taken at 4, 8, and 12 h after the treatment and stored at −80°C. RNA was extracted from the already stored samples at −80°C with the E.Z.N.A^®^ plant RNA extraction kit (Omega Bio-tek, Inc., Norcross, GA, United States) using the given protocol. Five hundred nanograms of RNA of each sample was taken for cDNA synthesis by EasyScript^®^ One-Step gDNA Removal and cDNA Synthesis SuperMix (TransGen Biotech., Shanghai, China) following the kit instruction. The cDNA samples were diluted 25 times with double-distilled water. The quantitative real-time PCR (RT-qPCR) was prepared according to the protocol of QuantiNova SYBR Green PCR Master Mix (QIAGEN, Pudong, Shanghai, China) and run on a Light Cycler^®^96 (Roche Diagnostics, Indianapolis, IN, United States). The RT-qPCR program was set as follows: 95°C for 10 min, 95°C for 10 s, 60°C for 20 s, and 72°C for 20 s (45 cycles); 95°C for 2 min, 60°C for 30 s, an then stimulation to 95°C. The calculation of the relative gene expression was done by the 2^–ΔΔ*Ct*^ method as described previously (Livak and Schmittgen, 2001).

### Statistical Analysis

Statistical analyses were done in R Studio 1.1.442. All data are presented as mean ± standard deviation. We analyzed the data from different treatments separately. The significant difference between treatments was tested by one-way analysis of variance (ANOVA). Tukey’s HSD test was used to determine the significant differences between pairs of means at *P* < 0.05.

## Results

### KAR^1^ Recovered the Inhibition of Seed Germination Caused by Osmotic and Salt Stresses

**FIGURE 1:**
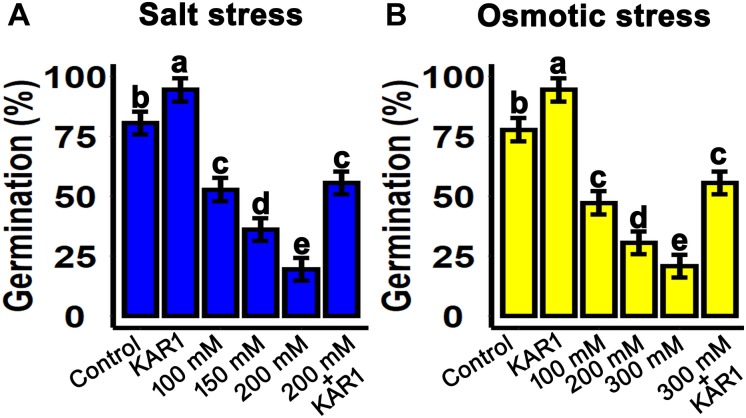
KAR^1^ promoted seed germination under salinity and osmotic stresses. **(A)** Salt stress was applied by supplementation of 100, 150, and 200 mM NaCl in 1/2 MS medium. KAR^1^ improved seed germination under salt stress (1 nM KAR^1^ + 200 mM NaCl + 1/2 MS). **(B)** Osmotic stress was applied by supplementation of 100, 200, and 300 mM NaCl in 1/2 MS medium. KAR^1^ improved seed germination under osmotic stress (1 nM KAR^1^ + 300 mM Mannitol + 1/2 MS). All data were collected 10 days after seed germination and analyzed by one-way ANOVA. Multiple comparisons were made by Tukey’s HSD test at *P* < 0.05 significance level (*n* = 5).

Salt and drought stresses are the crucial factors inhibiting seed germination. In this experiment, the salt and osmotic stresses were mimicked by sodium chloride (NaCl) and mannitol, respectively. The seeds were sown on 1/2 MS medium supplemented with 100, 150, and 200 mM NaCl. The results showed that seed germination was inhibited with increasing NaCl concentrations. Nevertheless, supplementation with 1 nM KAR^1^ in 1/2 MS + 200 nM NaCl medium resulted to a significant recovery in seed germination (Figure 1A). Similarly, under osmotic stress, seed germination was significantly repressed with increasing mannitol concentrations from 100 to 300 mM in 1/2 MS medium. Seed germination was also improved when 1 nM KAR^1^ was supplemented in 300 mM mannitol medium (Figure 1B). These results suggested that karrikins have the potential to allow the recovery of seed germination under abiotic stresses.

### KAR^1^ Promoted Seedling Growth Under Salt and Osmotic Stresses

**FIGURE 2:**
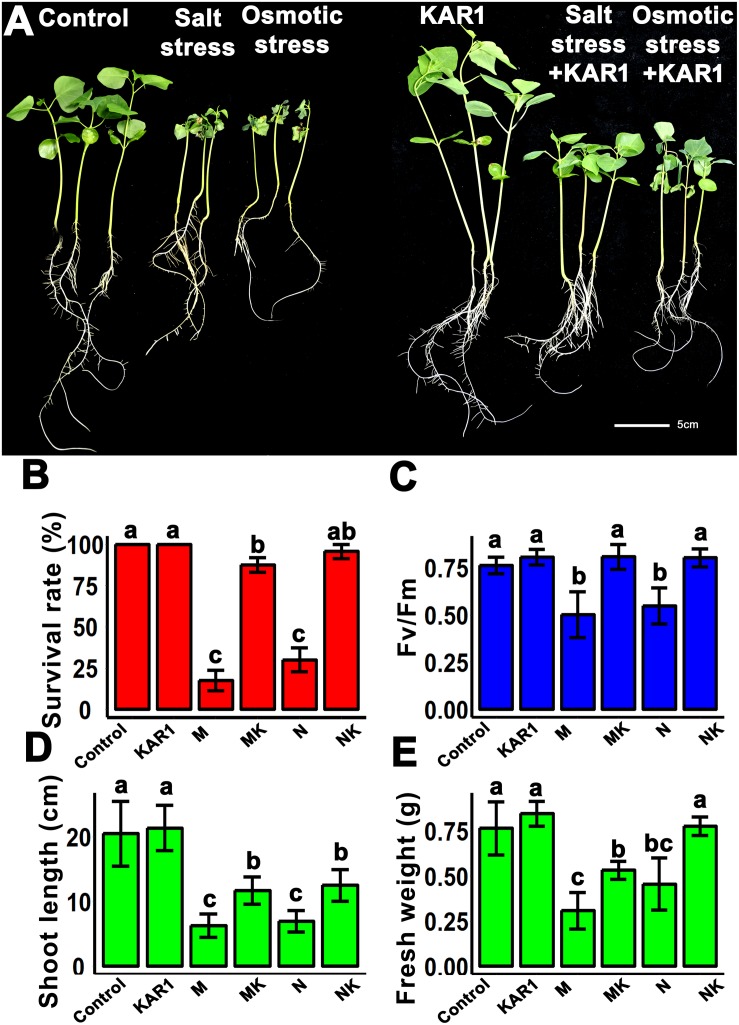
KAR^1^ alleviated salinity and osmotic stresses. **(A)** KAR^1^ improved seedlings growth under salt and osmotic stress, five-day-old seedlings were transplanted into 1/5 Hoagland plant-growing medium containing 1 nM KAR^1^, after 7 days of transplanting, salinity (mimicked by 150 mM NaCl), and osmotic stress (mimicked by 200 mM mannitol) were applied. Photographs were taken after 12 days of abiotic stresses. **(B)** Statistical presentation of survival rate after 10 days of salt and osmotic stress (*n* = 150). **(C)** Photochemical efficiency of the PSII (*n* = 15). **(D)** Shoot length (*n* = 15). **(E)** Fresh weight (*n* = 15) of seedlings. All data were analyzed by one-way ANOVA, and multiple comparisons were made by HSD Tukey’s test at *P* < 0.05 significance level. In **(B–E)**, Control, only 1/5 Hoagland nutrient solution, KAR^1^, 1 nM KAR^1^ in 1/5 Hoagland nutrient solution M, 150 mM mannitol in 1/5 Hoagland nutrient solution. MK, 150 mM mannitol + 1 nM KAR^1^ in 1/5 Hoagland nutrient solution. N, 150 mM NaCl in 1/5 Hoagland nutrient solution. NK, 150 mM NaCl + 1 nM KAR^1^ in 1/5 Hoagland nutrient solution.

Healthy seedlings are highly recommended to get vigorous and high-yielding mature plants. The results of this study showed that the KAR^1^ application improved seedling growth under salt and osmotic stresses (Figure 2A). Under salt and osmotic stresses, the survival rate of the seedlings was 30 ± 5 and 10 ± 5 (%), respectively. Interestingly, the plant survival rate was increased to 90 ± 5 and 80 ± 10 (%) by the addition of KAR^1^, separately under salt and osmotic stresses (Figure 2B). The supplementation of 1 nM KAR^1^ also increased the plant height, the maximum photochemical efficiency of PSII (Fv/Fm), and the biomass accumulation under both salt and osmotic stresses (Figures 2C–E), suggesting that karrikins are potent growth promoters in plants under salt and osmotic stresses.

### KAR^1^ Improved Root Growth Under Salt and Osmotic Stresses

**FIGURE 3:**
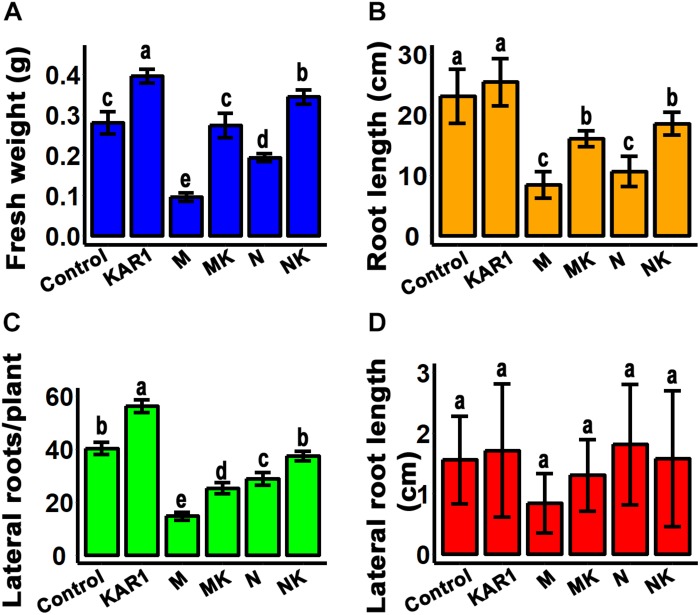
KAR^1^ promoted root growth under salinity and osmotic stresses. **(A)** Root fresh weight, **(B)** root length, **(C)** number of lateral roots per plant, and **(D)** lateral root length. All data were taken after 12 days after the application of abiotic stresses. All data (*n* = 15) were analyzed by one-way ANOVA, and multiple comparisons were made by Tukey’s HSD test at *P* < 0.05 significance level. Control, only 1/5 Hoagland nutrient solution; KAR^1^, 1 nM KAR^1^ in 1/5 Hoagland nutrient solution; M, 150 mM mannitol in 1/5 Hoagland nutrient solution; MK, 150 mM mannitol + 1 nM KAR^1^ in 1/5 Hoagland nutrient solution; N, 150 mM NaCl in 1/5 Hoagland nutrient solution; NK, 150 mM NaCl + 1 nM KAR^1^ in 1/5 Hoagland nutrient solution.

Plant roots are the organs which initially respond to most of the abiotic stresses (Cavalcanti et al., 2007). Abiotic stress inhibits the taproot and lateral root growth in plants. The stress-tolerant plants could regulate their taproot and lateral root growth to adapt to the abiotic stresses (Robin et al., 2016). In this study, we discovered that KAR^1^ improved the root growth, resulting in significantly increased weight and taproot length, specifically under abiotic stress (Figures 3A,B). KAR^1^ treatment increased the number of lateral roots per plant, while the length of the lateral roots was not significantly affected (Figures 3C,D). The results suggested that karrikins improved the root growth, which contributed to increased osmotic and salt stress tolerance in *S. sebiferum* seedlings.

### The Core Metabolites Involved in KAR^1^-Induced Osmotic and Salt Stress Tolerance in *S. sebiferum*

**FIGURE 4:**
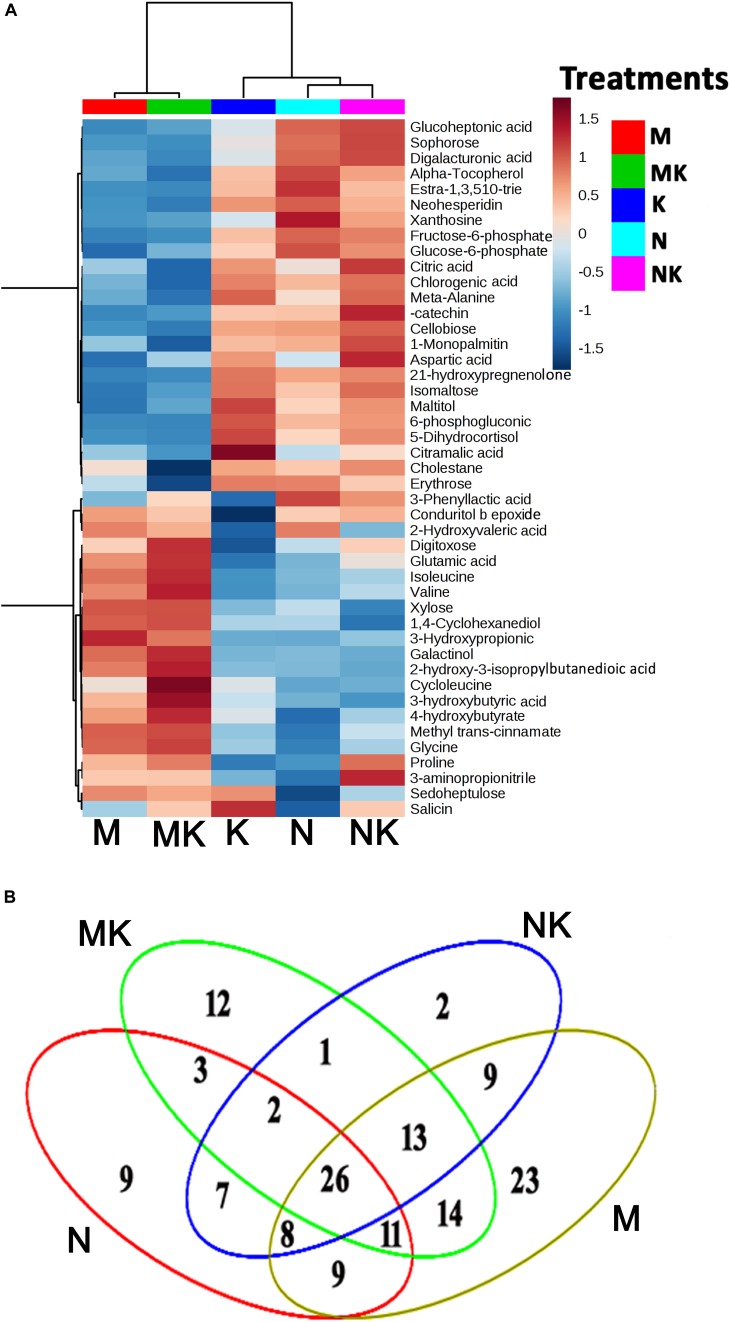
KAR^1^ regulated the metabolites in *S. sebiferum* under salt and osmotic stresses. **(A)** Heatmap hierarchical clustering of detected metabolites. The hierarchical tree was drawn based on detected metabolites in the leaves of 15-day-old *S. sebiferum* seedlings (*n* = 3) supplemented with KAR^1^ under osmotic (mannitol, 200 mM) and salt (NaCl, 150 mM) stresses for 6 days. The columns correspond to the treatments, while the rows represent different metabolites. **(B)** Venn diagram depicting the shared and common DAMs between the different treatments. K, 1 nM KAR^1^; M, 200 mM mannitol; N, 150 mM NaCl; MK, 200 mM mannitol + 1 nM KAR^1^; NK, 150 mM NaCl + 1 nM KAR^1^.

In the present study, we profiled the metabolic changes in *S. sebiferum* seedlings supplemented with KAR^1^ under salinity and osmotic stresses. In total, we detected 218 metabolites in the metabolome analysis of all samples. Fold changes between treatments and control are provided in Supplementary Table 2. In KAR^1^-treated seedlings, carbohydrates and polyols, such as digitoxose, xylose, galactinol, and sophorose, were increased under osmotic stress, while KAR^1^ induced xylose, cellobiose, digitoxose, isomaltose, glucose-6-phosphate, diglacturonic acid, and maltitol under salt stress. In addition, three organic acids such as 4-hydroxibutarate, 3-hydroxyburic acid, and 2-hydroxivaleric acid were induced by KAR^1^ under osmotic stress, while KAR^1^ increased the levels of glucoheptonic acid, citric acid citramalic acid, and chlorogenic acid under salt stress. Meanwhile, the level of amino acids such as valine, proline, and glutamic acid were induced in KAR^1^-supplemented seedlings under osmotic and salt stresses. Moverover, KAR^1^ increased the levels of glycine, isoleucine, and cycloleucine under osmotic stress and beta-alanine, aminopropionitrile, and aspartic acid under salt stress (Figure 4A). The Venn diagram presents the shared and specific DAMs in different treatments under osmotic and salt stresses. We identified 22 metabolites in the KAR^1^ + NaCl treatment and 49 metabolites in the KAR^1^ + mannitol treatment, compared with those in the NaCl or mannitol treatments (Figure 4B).

### KAR^1^ Reduced Hydrogen Peroxide, Malondialdehyde Level, and Electrolyte Leakage Under Abiotic Stresses

**FIGURE 5:**
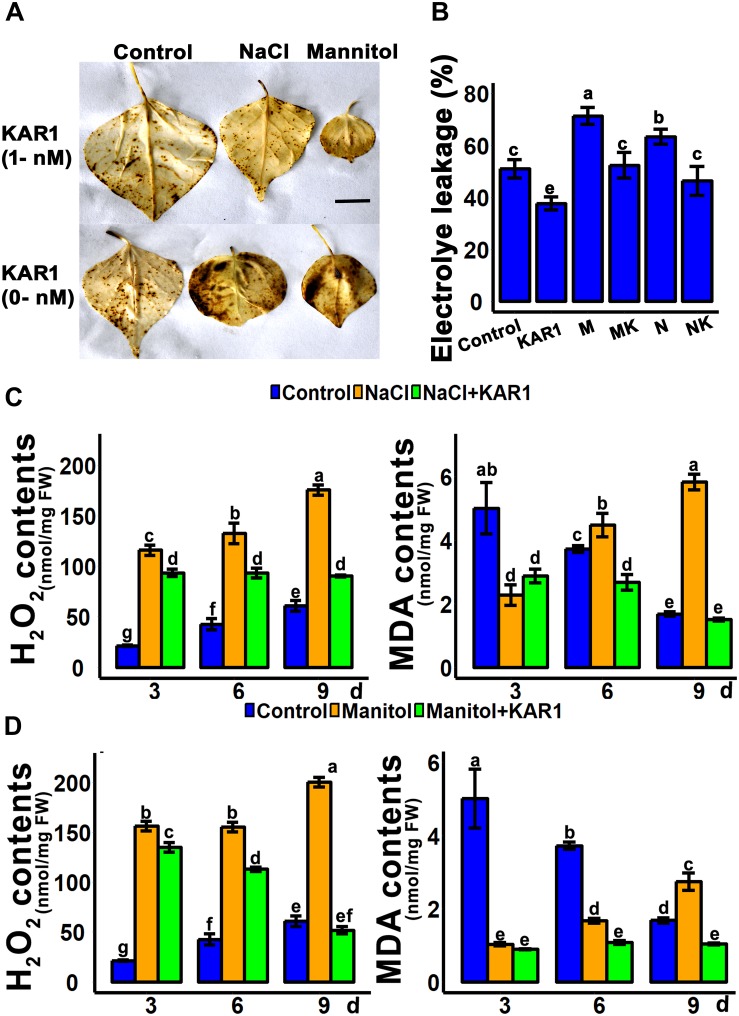
KAR^1^ reduced the H_2_O_2_ and MDA levels under abiotic stresses. **(A)** DAB staining of H_2_O_2_, **(B)** electrolyte leakage, **(C)** H_2_O_2_ contents and MDA contents under salt stress, and **(D)** H_2_O_2_ contents and MDA contents under osmotic stress. Five-day-old seedlings were transplanted into 1/5 Hoagland plant-growing medium containing 1 nM KAR^1^, after 10 days of transplanting, seedlings were subjected to salinity (mimicked by 150 mM NaCl), and osmotic stress (mimicked by 200 mM mannitol). The samples were taken randomly from the roots of five plants of each treatment. All data were analyzed by one-way ANOVA, and multiple comparisons were performed by HSD Tukey’s test at *P* < 0.05 significance level (*n* = 5). In the *x*-axis of each graph, “h” represents time in hours under abiotic stress.

**FIGURE 6:**
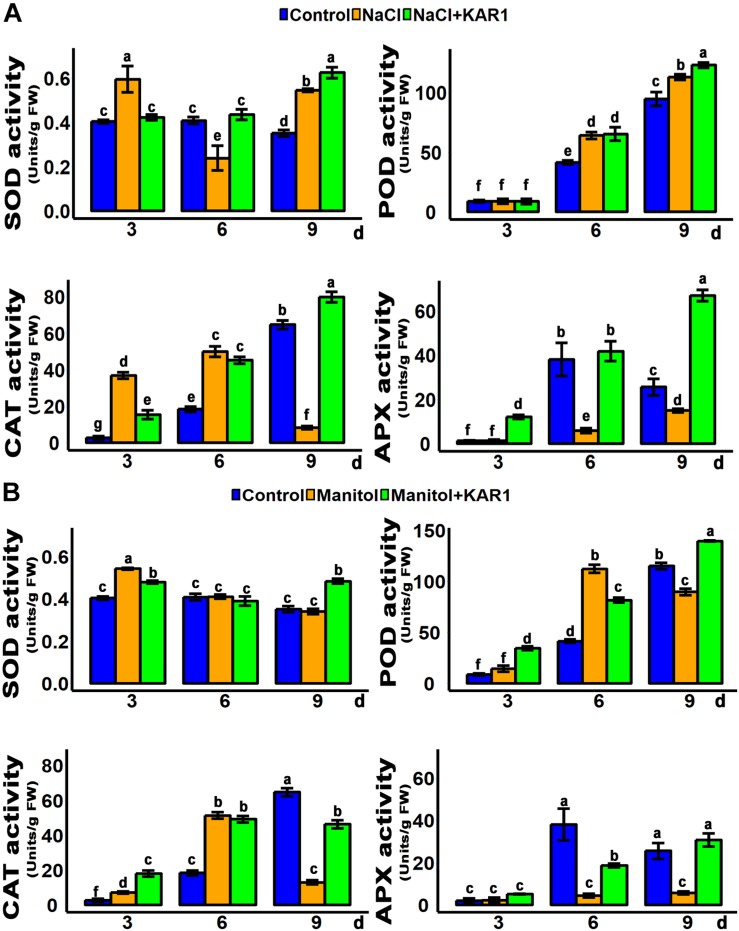
Anti-oxidant activity was increased in KAR^1^-treated seedlings. **(A)** SOD, POD, CAT, and APX activity under salt stress. **(B)** SOD, POD, CAT, and APX activity under osmotic stress. Five-day-old seedlings were transplanted into 1/5 Hoagland plant-growing medium containing 1 nM KAR^1^, after 10 days of transplanting, seedlings were subjected to salinity (mimicked by 150 mM NaCl), and osmotic stress (mimicked by 200 mM mannitol). The samples were taken randomly from the roots of five plants of each treatment. All data were analyzed by one-way ANOVA, and multiple comparisons were performed by HSD Tukey’s test at *P* < 0.05 significance level (*n* = 5). In the *x*-axis of each graph, “h” represents time in hours under abiotic stress.

Hydrogen peroxide is the main peroxidative molecule which could induce cell necrosis under environmental stresses (Ayala et al., 2014). Results showed that the endogenous H_2_O_2_ level was much lower in the KAR^1^-treated seedlings under abiotic stresses (Figures 5C,D). MDA, an end product of lipid peroxidation, is a biochemical marker for the measurement of cell epidermal layer degradation (Nita and Grzybowski, 2016). The MDA level was increased in plant leaves under salt and osmotic stresses, but KAR^1^ supplementation reduced the level of MDA (Figures 5C,D). The DAB staining results showed that KAR^1^ reduced the level of H_2_O_2_ in *S. sebiferum* leaves under salt and osmotic stresses (Figure 5A). Furthermore, the results also showed that the KAR^1^ application reduced the electrolyte leakage under abiotic stresses (Figure 5B). Karrikins are reported to enhance the antioxidant level in the seed germination of *Eragrostis tef* (Ghebrehiwot et al., 2008), and antioxidants such as ascorbate peroxidase, catalase, peroxidase, and superoxide dismutase protect cell apoptosis by scavenging the ROS and alleviating the biotic and abiotic stresses. We further investigated the enzymatic activity of these antioxidants with or without KAR^1^ under abiotic stresses. The results showed that the levels of all antioxidants were significantly increased in KAR^1^-treated seedlings under salt and osmotic stresses (Figures 6A,B), suggesting that karrikins conferred the abiotic stress *via* promoting the antioxidant levels. Overall, these results suggested that karrikins could control the endogenous H_2_O_2_ level, prevent the electrolyte leakage, and improve the membrane integrity under abiotic stresses.

### Karrikin-Responsive Gene Expression Was Increased Under Salt and Osmotic Stresses

**FIGURE 7:**
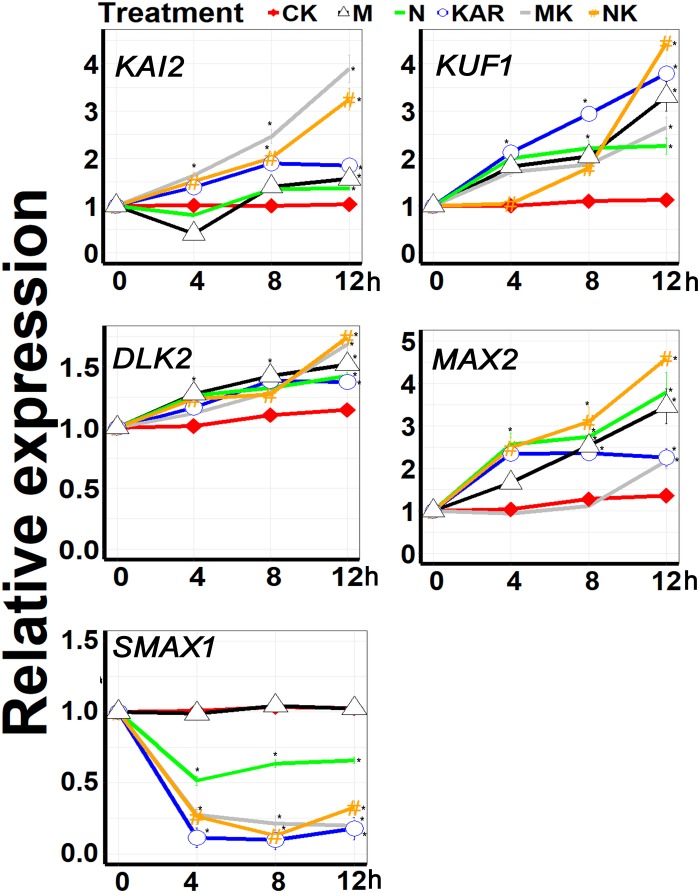
KAR^1^ promoted the expression of the key abiotic stresses acclimation-related genes. The expression of genes involved in abiotic stress acclimation was investigated in three separate plants of each line after 4, 8, and 12 h of osmotic and salt treatment. Five-day-old seedlings were transplanted into 1/5 Hoagland plant-growing medium containing 1 nM KAR^1^; after 10 days of transplanting, the seedlings were subjected to salinity (mimicked by 200 mM NaCl) and osmotic stress (mimicked by 200 mM mannitol). The samples were taken randomly from the roots of three plants of each treatment. *S. sebiferum UBQ10* was used as a reference gene; control treatment at 0 h was defined as 1. All data were analyzed by one-way ANOVA, and multiple comparisons were performed by Tukey’s HSD test at *P* < 0.05 significance level (*n* = 3). For all data, values significantly different from the control are marked with asterisks (*), and a common asterisk is shown at merged points in the graph. In the *x*-axis of each graph, “h” represents time in hours under abiotic stress. CK, control; KAR, 1 nM KAR^1^; M, 200 mM mannitol; N, 200 mM NaCl; MK, 200 mM mannitol + 1 nM KAR^1^; NK, 200 mM NaCl + 1 nM KAR^1^ supplementation in 1/5 Hoagland nutrient solution.

*KAI2* and *MAX2* are key signaling components of karrikins, which were recently reported to be involved in abiotic stresses, such as drought and salt stresses (Li et al., 2017; Wang et al., 2018). Other genes, such as *DLK2*, *KUF1*, *SMAX1*, and *SMAX2* are also karrikin-responsive genes, but whether they also play a role in abiotic stress responses remains elusive. In this study, we found that the expression of *KAI2*, *MAX2*, *DLK2*, and *KUF1* was upregulated under abiotic stresses (Figure 7). Exogenous KAR^1^ supplementation further boosted the expression level of *KAI2*, *MAX2*, *DLK2*, and *KUF1* under both osmotic and salt stresses. Karrikins signaling gene *SMAX1* was downregulated under abiotic stresses, and KAR^1^ supplementation further decreased the expression levels of *SMAX1* (Figure 7). The results suggested that karrikins might confer abiotic stresses by targeting the specific signaling components.

### KAR^1^ Affected ABA-Related Gene Expression and Reduced Endogenous ABA Contents in *S. sebiferum* Under Salt and Osmotic Stresses

**FIGURE 8:**
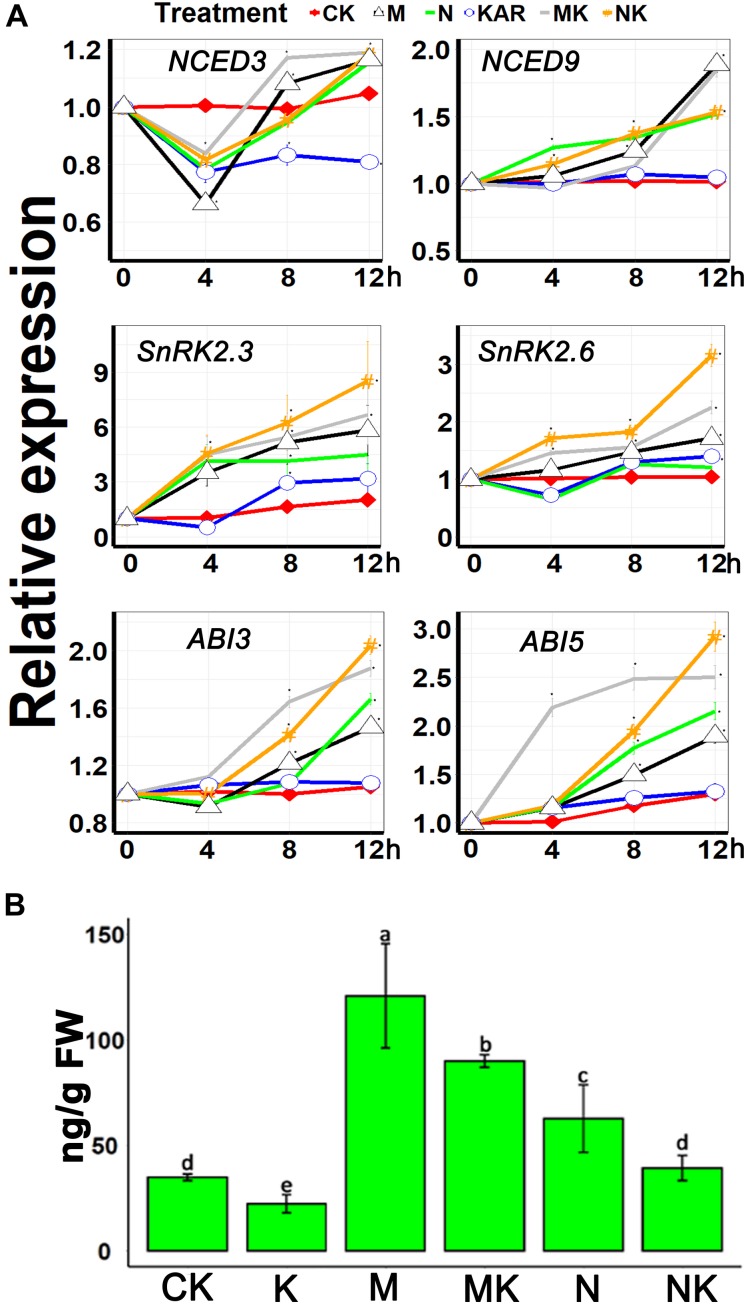
KAR^1^ regulated the ABA-related gene expression and ABA concentration under abiotic stresses. **(A)** The expression of genes involved in ABA biosynthesis and signaling was investigated in three separate plants of each line after 4, 8, and 12 h of osmotic and salt treatment. Five-day-old seedlings were transplanted into 1/5 Hoagland plant-growing medium containing 1 nM KAR^1^; after 10 days of transplanting, the seedlings were subjected to salinity (mimicked by 200 mM NaCl) and osmotic stress (mimicked by 200 mM mannitol). The samples were taken randomly from the roots of three plants of each treatment. **(B)** ABA concentration in the leaves of 15-day-old *S. sebiferum* seedlings after 6 days of osmotic and salt stresses. *S. sebiferum UBQ10* was used as a reference gene; control treatment at 0 h was defined as 1. All data were analyzed by one-way ANOVA, and multiple comparisons were performed by Tukey’s HSD test at *P* < 0.05 significance level (*n* = 3). For all data, values significantly different from the control are marked with asterisks (*), and a common asterisk is shown at merged points in the graph. CK, control; KAR, 1 nM KAR^1^; M, 200 mM mannitol; N, 200 mM NaCl; MK, 200 mM mannitol + 1 nM KAR^1^; NK, 200 mM NaCl + 1 nM KAR^1^ supplementation in 1/5 Hoagland nutrient solution. In the x-axis of each graph, “h” represents time in hours under abiotic stress.

*NINE-CIS-EPOXYCAROTENOID DIOXYGENASE 3* (*NCED3*) and *NCED9* are the essential genes involved in the ABA biosynthesis (Qin and Zeevaart, 1999; Seo et al., 2004; Urano et al., 2009). Our results showed that osmotic and salt stresses induced the expression of *NCED3* and *NCED9*, but KAR^1^ supplementation did not significantly upregulate the expression of *NCED3* and *NCED9* under abiotic stresses (Figure 8A). The class III *SNF-1-related protein kinases 2* (*SnRK2s*) are involved in the regulation of ABA signaling (Hiroaki et al., 2007). It has been discovered that SnRK2s are responsive to abiotic stresses (Kulik et al., 2011). The results of this study showed that KAR^1^ supplementation significantly increased the expression of *SnRK2.3* and *SnRK2.6* under abiotic stresses (Figure 8A). *ABA Insensitive 3* (*ABI3*) and *ABA Insensitive 5* (*ABI5*) are the ABA signaling genes which function together to ensure an adequate response to adverse conditions (Nakamura et al., 2001; Skubacz et al., 2016). KAR^1^ supplementation also induced the expression of *ABI3* and *ABI5* under abiotic stresses (Figure 8A). In addition, we identified that the endogenous ABA level was higher in NaCl- or mannitol-treated seedlings as compared with the KAR1 + NaCl (mannitol)-treated seedlings (Figure 8B). These results suggested that karrikins might induce tolerance to the abiotic stresses *via* the regulation of the ABA signaling pathway.

### KAR^1^ Induced Abiotic Stress-Related Gene Expression Under Salt and Osmotic Stress

**FIGURE 9:**
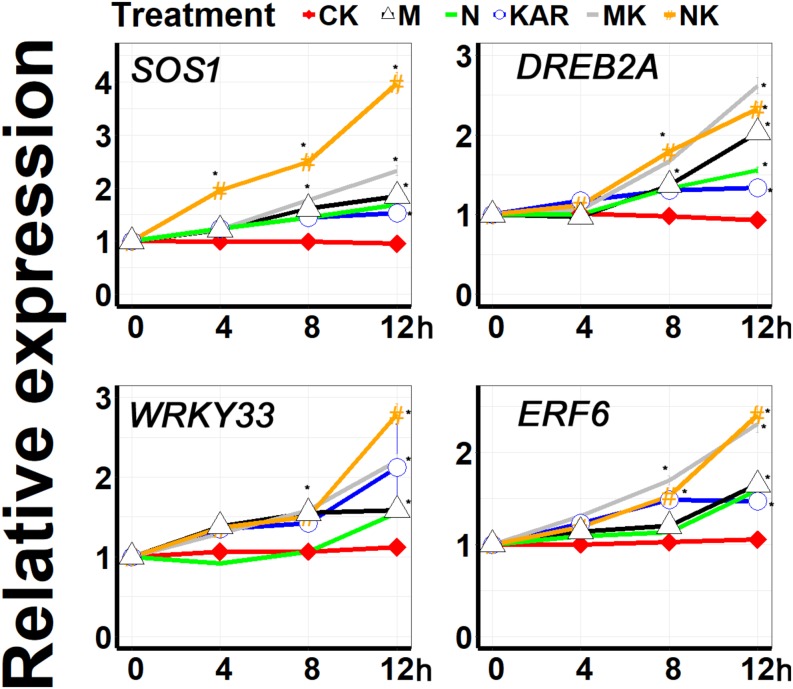
The expression of karrikin-responsive genes was induced by KAR^1^ under abiotic stresses. The expression of gene response to karrikins was investigated in three separate plants of each line after 4, 8, and 12 h of osmotic and salt treatment. Five-day-old seedlings were transplanted into 1/5 Hoagland plant-growing medium containing 1 nM KAR^1^; after 10 days of transplanting, the seedlings were subjected to salinity (mimicked by 200 mM NaCl) and osmotic stress (mimicked by 200 mM mannitol). The samples were taken randomly from the roots of three plants of each treatment. *S. sebiferum UBQ10* was used as a reference gene; control treatment at 0 h was defined as 1. All data were analyzed by one-way ANOVA, and multiple comparisons were performed by Tukey’s HSD test at *P* < 0.05 significance level (*n* = 3). For all data, values significantly different from the control are marked with asterisks (*), and a common asterisk is shown at merged points in the graph. In the *x*-axis of each graph, “h” represents time in hours under abiotic stress. CK, control; KAR, 1 nM KAR^1^; M, 200 mM mannitol; N, 200 mM NaCl; MK, 200 mM mannitol + 1 nM KAR^1^; NK, 200 mM NaCl + 1 nM KAR^1^ supplementation in 1/5 Hoagland nutrient solution.

Plants have developed different kinds of coping mechanisms for survival under harsh conditions. At the molecular level, *SALT OVERLY SENSITIVE* (*SOS*), *WRKY*, *DREB*, and *ERF* are the key gene families involved in the regulation of stress response and adaptation in plants. A large number of molecular and genetic investigations revealed that *WRKY33* transcription factor plays a crucial role in abiotic stress responses (Jiang and Deyholos, 2009; He et al., 2016). *DREB2A*, which encodes a DRE-binding protein, is involved in dehydration and salt and promote the abiotic stress-responsive gene expression (Nakashima et al., 2000). *SOS1* plays a key role in the regulation of salt tolerance (Zhu, 1997; Zhu et al., 1998; Shi et al., 2000). *ERF6* was found to be involved in alleviating abiotic stresses *via* regulating ROSs and stress-related genes (Nasser et al., 2013; Warmerdam et al., 2019). In this study, KAR^1^ significantly enhanced the expression level of *WRKY33* under abiotic stresses (Figure 9). KAR^1^ could highly induce the expression of *DREB2A* and *SOS1* under salt and osmotic stress, indicating that karrikins might regulate salt stress tolerance *via* inducing the expression level of the *SOS1* gene (Figure 9). Under abiotic stresses, KAR^1^ significantly upregulated the expression of *ERF6* (Figure 9). Collectively, these results revealed that karrikins could directly target the stress-responsive genes in the regulation of stress adaptation in *S. sebiferum* seedlings.

## Discussion

Karrikins, which are derived from the burning smoke of plant biomass, can enhance the seed germination of many plant species. Karrikins are also involved in the regulation of hypocotyl development and photomorphogenesis (Jain and Van, 2007; Nelson et al., 2010). Recently, it has also been discovered that karrikins are involved in different abiotic stresses alleviation in different plant species (Ghebrehiwot et al., 2008; Sunmonu et al., 2016). Nevertheless, the mechanism of karrikins in the regulation of stress adaptation remains largely elusive. In this study, we found that KAR^1^ improved seed germination and seedling growth under osmotic and salt stresses in the oil plant *S. sebiferum*. KAR^1^ also significantly induced the accumulation of stress-related secondary metabolites and the expression of stress-responsive genes and ABA signaling genes, which contributed to the improved stress tolerance in *S. sebiferum* seedlings.

Abiotic stresses, especially osmotic and salt stresses, are critical factors affecting seed germination in many crops and tree species. In *S. sebiferum*, we found that seed germination was reduced with salt and osmotic stress stimulation, which is in agreement with previous reports in *Phaseolus* species and tomato (Bayuelojimenez et al., 2002; Jain et al., 2006). In this study, supplementation of KAR^1^ recovered seed germination under abiotic stresses (Figure 1). Previously, KAR^1^ was used to promote seed germination and seedling vigor in tomato under salt (Jain and Van, 2007). In maize, the smoke (containing karrikins) was proven to alleviate the salt stress resistance and improved seed germination, suggesting that compounds present in smoke water could regulate some physiological process and also control the ion homeostasis (Iqbal et al., 2013). It has been reported that karrikins improved seed germination and seedling growth in *E. tef* under osmotic and heat stresses (Ghebrehiwot et al., 2008). Karrikins could promote seed germination by upregulating the GA biosynthesis genes, activating ROS-scavenging antioxidants, and mobilizing soluble sugars in seeds (Nelson et al., 2008; Sunmonu et al., 2016). However, in *Arabidopsis*, karrikin treatment negatively regulates seed germination under various stresses (Wang et al., 2018). These results suggested that karrikins may play diverse roles in the regulation of seed germination in response to the abiotic stresses in different species. Our results were in accord with that in tomato and maize (Jain et al., 2006; Iqbal et al., 2013; Sunmonu et al., 2016), suggesting that KAR^1^ is a potent compound which promoted seed germination under osmotic and salt stresses.

Salt and osmotic stresses cause a high rate of seedling mortality and stunted growth in different plant species. The results showed that salt and osmotic stresses reduced the root and shoot growth as well as caused mortality in *S. sebiferum* seedlings. The improvement of root growth by KAR^1^ in the seedlings might provide strength to the seedling against the stresses. Roots are the plant organs susceptible to the surrounding environmental changes. The response of the root system to abiotic stress, such as salt and drought, can be very dynamic and complex. Under salt and osmotic stresses, water potential occurred in roots, which resulted in reduced root and shoot growth and also caused a reduction in cell wall synthesis and inhibition of cell expansion (Chaitanya et al., 2003; Robin et al., 2016). Our results suggested that KAR^1^ promoted the main root growth and significantly increased the lateral root number (Figure 3), suggesting a significant protective role of karrikins in the regulation of root growth under adverse environmental stresses.

The metabolites are the intermediates or the end products of metabolism, playing significant physiological and biochemical roles in plants (Luo, 2015). It is estimated that the total number of plant metabolites exceeds 200,000 (Pichersky et al., 2006), reflecting their multifaceted functions in the plant’s life cycle. As our results already showed that KAR^1^ could significantly improve the stress adaptation of the *S. sebiferum* seedlings under osmotic and salt stresses, the metabolic changes of the KAR^1^-treated plants subjected to the stresses could provide novel insights into the mechanisms of KAR^1^-regulated stress adaptation. In the present investigation, the levels of numerous metabolites were altered, offering an excellent opportunity to identify novel KAR^1^-responsive compounds under osmotic and salt stresses. Our observations are in perfect agreement with the study of Khan et al. (2019), who observed that plant growth regulators regulated many metabolites under abiotic stresses. In this study, KAR^1^ induced the accumulation of many carbohydrates, organic acids, and amino acids under osmotic and salt stresses (Figure 4A). Similarly, Kim et al. (2007) and Guo et al. (2018) revealed that osmotic and salt stresses affect the amino acid biosynthesis, glycolysis, and sucrose metabolism in *Arabidopsis*. Amino acids, such as glycine, valine, and glutamic acid, are involved in the scavenging of ROSs under abiotic stresses (Einset and Connolly, 2009; Chen and Heuer, 2013; Igamberdiev and Eprintsev, 2016). Organic acids, such as citric acid, chlorogenic acid, and aconitic acid, are important players in the maintenance of redox balance, production, scavenging, and consumption of ATP, in the support of protonic and ionic gradients on membranes, and in the acidification of extracellular spaces (Igamberdiev and Eprintsev, 2016). Proline is also an essential variable amino acid in determining protein and membrane structures and regulates the stabilization of various antioxidant enzymes, which contributes to the maintenance of the intracellular redox homeostasis (Liang et al., 2013). In this study, the metabolome analysis is suggesting that KAR^1^-induced accumulation of some amino acids and organic acids, which have a critical role in the redox reaction, contributes to the regulation of redox homeostasis by KAR^1^ in response to drought and osmotic stresses.

The abiotic stresses could induce a significant increase of ROS, over-accumulation of which could cause severe cellular oxidative damage. ROS cause oxidation of membrane lipids, which leads to degradation of the cell membrane. The breakage of membranes, due to lipid peroxidation, is considered as one of the most destructive cellular processes and a marker of cell damage under different stresses (Ayala et al., 2014). MDA, an end product of lipid peroxidation, together with hydrogen peroxide, is considered as an essential marker of cell damage and necrosis in living organisms (Nita and Grzybowski, 2016). Under abiotic stresses, cell membrane breakage could also cause leaking electrolyte from the cytosol that may result in the death of the plant (Demidchik et al., 2014). This study found that MDA and H_2_O_2_ contents were significantly lower in KAR1-treated plants under osmotic or salt stresses (Figure 5). Previous reports showed that stress-resistant plants produce a higher dose of enzymatic antioxidants such as POD, SOD, APX, and CAT to eliminate or reduce the excess ROS. SOD converts superoxide to the less toxic H_2_O_2_ molecule, which is further detoxified into H_2_O by CAT, APX, and POD (Asada, 2000; Sairam et al., 2005; Cavalcanti et al., 2007; Chang et al., 2009; Mullineaux et al., 2010; Chen and Heuer, 2013; Del Río et al., 2018). KAR^1^-treated seedlings had increased levels of enzymatic antioxidants under osmotic and drought stresses (Figure 6), which may partly explain why KAR^1^-treated plants were more tolerant to abiotic stresses. Overall, the predominant increase of the antioxidants and the reduction of H_2_O_2_, MDA, and electrolyte leakage emphasized the role of karrikins in maintaining the redox homeostasis and in the prevention of oxidative damage during abiotic stress acclimation.

Karrikins signaling genes, such as *KAI2* and *MAX2*, have been reported to be involved in the alleviation of abiotic stresses (Bu et al., 2014; An et al., 2016; Li et al., 2017; Wang et al., 2018, 2019). Karrikins signaling genes are also reported to enhance the sensitivity to ABA in *Arabidopsis* and regulate ABA biosynthesis and signaling genes (Bu et al., 2014; Li et al., 2017; Wang et al., 2018, 2019). ABA is an essential phytohormone in the regulation of abiotic stress adaptation (Xuexuan et al., 2010; Lee and Luan, 2012). In this study, the results showed that KAR^1^ significantly reduced the ABA level, under osmotic or salt stress, and several ABA signaling genes, such as *ABI3* and *ABI5*, were differentially regulated by KAR^1^ treatment (Figures 8A,B). The previous report showed that *KAI2* transgenic *Arabidopsis* inhibited seed germination and induced the stomata closure when exposed to exogenous ABA (Li et al., 2017). ABA stimulates several changes in plant physiological, molecular, and developmental progressions, resulting in plant adaptation to the stress environment (Ton et al., 2009). Abiotic stresses can induce ABA biosynthesis, which further activates the expression of stress-related genes and stomatal closure (Lee and Luan, 2012). This study suggests that karrikins might have a direct interaction with the ABA regulatory genes in the regulation of stress adaptation.

Stress-resistant plants have developed a specific molecular mechanism to cope with undesirable conditions. There are specific genes such as *SOS1* (*Salt Overly Sensitive 1*), *DREB2A* (*Dehydration-responsive Element-Binding Protein 2A*), *ERF6* (*Ethylene Reception Factor 6*), and *WRKY33*, which were previously reported as osmotic and salt stress regulatory genes (Shi et al., 2000; Jiang and Deyholos, 2009; Nasser et al., 2013; Nakashima et al., 2014; El-Esawi et al., 2017; Warmerdam et al., 2019). Previous reports showed that the overexpression of *TaWRKY33* led to significant drought and heat tolerance in *Arabidopsis* (He et al., 2016). *Arabidopsis DREB2* genes were found to be involved in osmotic and salt stress resistance and regulate the expression of stress-responsive genes (Nakashima et al., 2000). The SOS family proteins were reported as salt stress regulatory proteins in *Arabidopsis* (Zhu, 1997; Shi et al., 2000). Ethylene response factor 6 was found to be involved in abiotic stress resistance *via* the regulation of ROS signaling in *Arabidopsis* (Nasser et al., 2013). In this study, KAR^1^ significantly upregulated these stress-related genes under osmotic and salt stresses (Figure 9), which was in agreement with a previous study that karrikins receptor gene *KAI2* could upregulate the expression of *DERB2A*, *EFR5*, and *WRKY33* under abiotic stresses (Wang et al., 2018), indicating that the karrikin-responsive genes might have direct interactions with the stress regulatory genes in *S. sebiferum*.

## Conclusion

**FIGURE 10:**
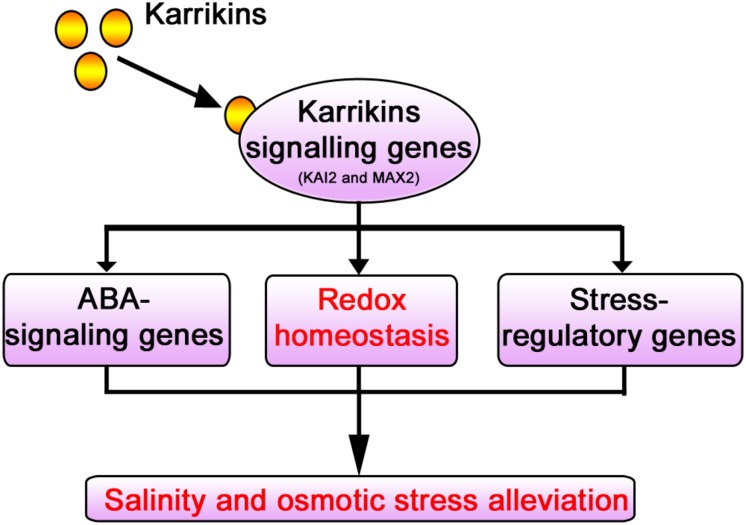
Model illustrating the mechanism of karrikins in the alleviation of abiotic stresses. Karrikins regulate the expression level of karrikins signaling genes (Nelson et al., 2010). Under abiotic stress, karrikins signaling genes such as *KAI2* and *MAX2* are involved in abiotic stress alleviation (Bu et al., 2014; An et al., 2016; Li et al., 2017; Wang et al., 2018, 2019). KAR^1^ (karrikin) regulates redox homeostasis (Figures 4, 6) and promotes the expression level of ABA-responsive genes and abiotic stress regulatory genes (Figure 8), which were previously reported to enhance drought and salt stress tolerance (Lee and Luan, 2012).

KAR^1^ supplementation at a nanomolar concentration significantly improved the stress tolerance during the seed germination and the seedling growth in *S. sebiferum*. Specifically, it promoted seedling growth and biomass accumulation and increased the primary root length and the number of lateral roots under salt and osmotic stresses. KAR^1^ induced many stress-related metabolites, such as sugars, organic acids, and amino acids, under abiotic stress. KAR^1^ application significantly promoted the activities of the antioxidants like SOD, POD, CAT, and APX, which led to a significant reduction of hydrogen peroxide, malondialdehyde, and electrolyte leakage under abiotic stresses. KAR^1^ also upregulated the stress regulatory-related and ABA signaling genes. Conclusively, this study suggests that karrikins can improve seed germination and seedling growth under salt and osmotic stresses *via* regulating redox homeostasis as well as by upregulating the ABA signaling and stress regulatory genes (Figure 10). This study clarified the stresses regulatory mechanism of karrikins at the physiological and molecular level, which will provide a foundation for further understanding of the role of karrikins in the regulation of abiotic stress tolerance in plants. This study also provides a novel clue for farmers and nursery growers in saline soil and arid area on how to promote the crop seedling’s growth under stressed conditions.

## Data Availability Statement

The datasets generated for this study can be found in the Supplementary Data 1.

## Author Contributions

FS, XW, JN, and LW designed the experiments. JN, FS, SH, JH, XW, WL, QW, DW, YY, and HH carried out the experiments. FS, WL, JN, XC, and CL analyzed the data and took photographs. FS and JN wrote the manuscript. All authors reviewed and approved the final manuscript.

## Conflict of Interest

The authors declare that the research was conducted in the absence of any commercial or financial relationships that could be construed as a potential conflict of interest.

## Footnotes

1 https://www.metaboanalyst.ca
