# Supplementary material for: Karrikin Improves Osmotic and Salt Stress Tolerance via the Regulation of the Redox Homeostasis in the Oil Plant Sapium sebiferum
Source: Front Plant Sci. 2020 Mar 24;11:216. doi: 10.3389/fpls.2020.00216 (PMC7105677; doi:10.3389/fpls.2020.00216)
Supplement: Supplementary file 1 [file Table_1.docx]

**Supplementary Table 1**

**List of qPCR primers**

| **Gene** | **Sense Primer (5'---------3')** | **Anti-sense Primer (5'---------3')** |
| --- | --- | --- |
| *SsKAI2* | CTATTGCTTCCATTTCTCGCCCAGATC | TCTCAAATCCTCCATAGTAATCCACATCGT |
| *SsMAX2* | TCTCTGCTTGTAAGAATCTCAATGCTGTAG | TTCAACCTGTATCCGAATCTCCTCCAA |
| *SsDLK2* | CAAGAGCCATGAACGCAAGAATCG | CCAAGCAGATTGATCTCCTCCATATCC |
| *SsKUF1* | AGTTCTCGGCGGATGTGGCTAA | AACATTCACCATACATAGTTTCCCTCTCC |
| *SsSMXL1* | ACAGTCTACTAATGCCAACAACTTCATCAG | TGTCCAATTCGAGCCTCACAACCAT |
| *SsSOS1* | GTGGTCTATCAGTTGTTCTATCGGATGG | CAGTGCAATCTCTATCACCGTGTCAT |
| *SsDREB2A* | CAGAGGTGTTAGACAACGGACATGG | AGCAGCATCATACGCAAGAGCAG |
| *SsWRKY33* | GGACAGCACAACCACCAACCTC | TCCAGCATCCTTCACACGCTTATTAG |
| *SsERF6* | ATCCAATTCACTAACTCCAACCAGCAA | GGCGGCTTCGATAGCAGAATCAA |
| *SSNCED3* | GGATTTGCCAAAGTAGACCTCTCAACT | CACAATCTTCATCTTCATCTCCTCTTCCA |
| *SsNCED9* | AGCAGGAGGAGGTGTGAGAATGG | AATGAAGAATTGAAGGAGTGTGGAGAGAG |
| *SsSnRK 2.3* | GTGTCCACTACTCAATTCCAGACTATGTT | GCAAGCCATCTTCCTCCACTTCC |
| *SsSnRK 2.6* | GAAGCCACCATACCAGCAGCAG | GTCCTCCTCCATGTCATCATCAATATCC |
| *SsABI3* | GTTGGTGACAGGCTGGTGAGATTAG | GCCATTCTCTTCTTCCTCGCTTCTT |
| *SsABI5* | GGCACGGTAGCAGTAGCAATGG | CGATGGCAATGAAGATGAAGAACGATG |
| *SsUBQ10* | CTTGCGTCTGCGTGGAGGTATG | GGCGAAGATCAATCTCTGCTGGTC |

All primers were selected from Open Reading Frame of all genes. Primer Premier 6.0 was used to design all primers. Full length sequences of all genes are given in Supplementary data 1.
