# Supplementary material for: Karrikin Improves Osmotic and Salt Stress Tolerance via the Regulation of the Redox Homeostasis in the Oil Plant Sapium sebiferum
Source: Front Plant Sci. 2020 Mar 24;11:216. doi: 10.3389/fpls.2020.00216 (PMC7105677; doi:10.3389/fpls.2020.00216)
Supplement: Supplementary file 2 [file Table_2.docx]

**Supplementary Table 2**

Fold changes of metabolites in *Sapium sebiferum* after six days of salt or osmotic stresses treatment. Fold changes were calculated using the formula log_2_^(Treatment/Control)^. K, 1 nM KAR^1^; M, 200 mM mannitol; N, 150 mM NaCl; MK, 200 mM mannitol+1 nM KAR^1^; NK, 150 mM NaCl+1 nM KAR^1^.

|  | Log_2_^(Treatment/Control)^ | | |  |  |
| --- | --- | --- | --- | --- | --- |
| Metabolites/Treatments | K | M | MK | N | NK |
| 2-hydroxypyridine | -0.92807 | -10.7405 | 2.02526 | 8.440411 | 0.202897 |
| Pyruvic acid | 2.149778 | -3.96564 | 1.149541 | 0.009368 | -0.34305 |
| lactic acid | -1.14308 | 0.784593 | 3.957592 | 0.116085 | -4.71519 |
| Glycolic acid | 0.080021 | -1.21113 | -0.42648 | 0.728036 | -0.17045 |
| Alanine | 1.29174 | -0.0939 | -0.63261 | 0.675738 | -2.24097 |
| Lactamide | 1.515859 | 1.025104 | -2.44756 | -2.60609 | 1.512686 |
| 3-Hydroxypropionic acid | -25.4715 | 33.95166 | 12.59887 | -18.5742 | -3.50476 |
| 3-Hydroxypyridine | 1.890699 | 6.944739 | -4.5422 | -7.69354 | 2.400302 |
| N-Methyl-DL-alanine | 1.727628 | -2.04511 | -1.00474 | -0.89834 | 1.220572 |
| Methyl Phosphate | -0.12763 | -0.97824 | 0.310086 | 0.546962 | -0.75118 |
| Succinate semialdehyde | 0.650262 | -2.9299 | -3.28871 | 2.889399 | 1.678948 |
| 2-Hydroxyvaleric acid | 1.69608 | -1.23717 | -0.74167 | -2.24451 | 1.527277 |
| Malonic acid | 0.027958 | -3.44881 | -0.15176 | 1.780838 | 0.791775 |
| Cycloleucine | -0.76859 | -2.86356 | 0.594357 | 1.037792 | 1 |
| Valine | 2.716702 | -2.77246 | -4.13415 | 2.235095 | 0.954813 |
| 1-Aminocyclopropanecarboxylic acid | -0.14235 | -0.29904 | -1.59683 | -0.7781 | 1.816313 |
| Naphthalene | 0.777988 | -2.10813 | -5.25802 | 1.576506 | 4.011662 |
| 4-hydroxybutyrate | -0.3029 | -0.61576 | -1.74474 | 1.681648 | -0.01825 |
| 2-Butyne-1,4-diol | -13.4361 | 28.50452 | 15.21729 | -8.01805 | -23.2677 |
| Carbobenzyloxy-L-leucine | -1.02479 | -0.46508 | -4.73018 | 2.646222 | 2.573834 |
| Dihydroxyacetone | 1.544444 | -0.80468 | 0.241718 | -0.78009 | -1.2014 |
| Oxamic acid | -0.25242 | -0.64907 | -0.44473 | 0.257677 | 0.088543 |
| Benzoic acid | 38.9074 | 62.15905 | 2.341427 | -49.947 | -54.4609 |
| Serine | 1.267919 | -4.76669 | -1.41063 | 2.76058 | 1.14882 |
| Ethanolamine | 0.901648 | -0.45224 | -1.71559 | 0.729489 | -0.46331 |
| Phosphate | 0.711304 | -0.3862 | -1.09493 | 0.670362 | -0.90054 |
| Glycerol | 1.802637 | -1.33816 | -4.51143 | 1.750668 | 1.296281 |
| 2-Deoxyuridine | -5.11875 | 0.310263 | -4.03607 | 5.711735 | 2.132826 |
| 2-Deoxyerythritol | -10.4003 | 15.55547 | 10.93442 | 0.559712 | -17.6493 |
| Isoleucine | 1.941196 | -2.27112 | -3.02383 | 1.393634 | 0.960121 |
| Threonine | 0.291815 | -0.9595 | -0.52735 | -0.12788 | 0.322918 |
| Proline | 1.015415 | -0.75294 | -0.96279 | 0.815381 | -1.11507 |
| Maleic acid | 5.68039 | -0.0418 | 2.078986 | -3.4535 | -5.26408 |
| Glycine | 0.301312 | -2.48772 | -2.22158 | 2.593987 | 0.814004 |
| 1,4-Cyclohexanedione | 0.284378 | -1.3631 | -1.4643 | 0.285021 | 1.257995 |
| Succinic acid | -0.589 | -0.04874 | -5.70127 | 1.215294 | 4.123719 |
| 2,2-Dimethylsuccinic Acid | 0.111148 | -3.44769 | 0.428719 | 1.145944 | 0.761875 |
| D-Glyceric acid | -0.32135 | -1.6596 | 0.853642 | 0.263477 | -0.13617 |
| Beta-hydroxypyruvate | 0.367148 | -0.28043 | -0.81238 | -0.49437 | 0.220023 |
| Uracil | 1.920135 | -0.72636 | -0.6799 | -0.46074 | -1.05314 |
| Itaconic acid | 1.320584 | -1.31282 | -1.67156 | 0.145762 | 0.518033 |
| Fumaric acid | 2.859333 | -0.71649 | -2.54556 | -1.01787 | 0.420576 |
| Serine | -0.99265 | 5.082405 | 1.015381 | -1.87938 | -4.22575 |
| 1-Indanol | 0.032433 | -0.29865 | -1.79167 | 0.633283 | 0.424601 |
| Pelargonic acid | 2.375766 | -2.59889 | -0.74466 | -1.24369 | 1.211478 |
| Tartronic acid | -2.28975 | 4.668769 | 8.212769 | -2.39417 | -9.19763 |
| L-Allothreonine | 0.29529 | 0.082517 | -1.14991 | 0.410375 | -0.63827 |
| cuminic alcohol | -0.51015 | -2.92163 | 1.03143 | 0.249919 | 1.150422 |
| 3-aminopropionitrile | 0.719316 | -0.56917 | -0.85022 | 1.091374 | -1.3913 |
| Glutaric Acid | -0.10258 | -0.6442 | 0.25322 | 0.544301 | -1.05073 |
| 2-methylfumarate | -0.06996 | -1.2292 | 1.154254 | -0.17136 | -0.68373 |
| Methyl trans-cinnamate | 0.076444 | -1.90891 | -1.7667 | 2.072223 | 0.526946 |
| N-Ethylglycine | 0.600633 | 0.275027 | -1.06047 | -0.86347 | 0.048274 |
| 6-hydroxy caproic acid | -3.93951 | 5.870147 | 3.506906 | -2.62823 | -3.80931 |
| Meta-Alanine | 2.359205 | -1.38389 | -3.21954 | 0.849017 | 0.395208 |
| Maleamate | 0.536243 | -0.92811 | -0.31363 | 0.087708 | -0.38221 |
| Erythrose | -0.64483 | -0.04299 | 0.556673 | -0.5189 | -0.34996 |
| 3-Aminoisobutyric acid | -5.63331 | -2.4868 | 1.636112 | 5.084331 | 0.399672 |
| Oxalacetic acid | 1.739199 | -0.44685 | 0.219584 | -2.08593 | -0.426 |
| Aminomalonic acid | -1.6968 | -6.42633 | 2.5518 | 4.739647 | -0.16831 |
| Citramalic acid | -1.41987 | 0.243458 | 0.34254 | 0.105792 | -0.27192 |
| Bis(2-hydroxypropyl)amine | -4.66695 | -2.80903 | -3.16359 | 10.76637 | -1.1268 |
| L-Malic acid | -0.52229 | -0.55433 | 0.211849 | -0.18332 | 0.048089 |
| Threitol | 4.645676 | 1 | -0.14928 | -3.375 | -3.1214 |
| 2-Hydroxyquinoline | -0.42912 | 0.103453 | -0.5731 | -0.22166 | 0.120435 |
| Asparagine | 35.02763 | 18.89687 | -15.9389 | -21.4101 | -17.5755 |
| 3-Hexenedioic acid | -0.88902 | 0.277985 | -1.70538 | 0.264276 | 1.052146 |
| Aspartic acid | 0.808063 | -1.53673 | -1.00802 | -0.2139 | 0.950587 |
| Methionine | 0.84273 | -3.2315 | 1.858773 | -1.11304 | 0.643032 |
| Oxoproline | 1.598385 | -0.89813 | -5.55057 | 0.415366 | 3.434947 |
| 4-aminobutyric acid | -5.82391 | -7.40086 | 8.519932 | 3.385418 | 0.319422 |
| L-glutamic acid | -0.21478 | -0.06521 | -1.06965 | -2.14798 | 2.497627 |
| 3-Isochromanone | -0.0521 | -0.58783 | -0.30868 | -0.19222 | 0.140836 |
| Pyrogallol | -0.69962 | 5.163264 | 0.997089 | -5.13706 | -1.32368 |
| Nornicotine | -0.70478 | 0.423213 | -0.26827 | -0.29645 | -0.15372 |
| 4-Hydroxyquinazoline | 2.820049 | -3.64055 | -1.28562 | 1.602289 | -0.49617 |
| Menthone | 1.230519 | -0.79975 | -0.60593 | -0.49141 | -0.33343 |
| Threonic acid | 0.493359 | -0.91969 | -0.83724 | 0.671119 | -0.40755 |
| N-Methyl-L-glutamic acid | 2.036422 | -0.56206 | -0.24601 | -1.51685 | -0.7115 |
| 2-hydroxy-3-isopropylbutanedioic acid | 8.006633 | -13.3517 | -19.249 | 10.86173 | 12.73226 |
| alpha-ketoglutaric acid | -0.49833 | 0.197369 | -0.01217 | -0.08199 | -0.60488 |
| MALONAMIDE | 0.218995 | -0.18431 | 0.720608 | -0.62344 | -1.13186 |
| 3-Phenyllactic acid | 1.677991 | 0.731915 | -0.5814 | -1.79673 | -1.03178 |
| 3-hydroxy-3-methylglutaric acid | 6.169568 | -5.31511 | 5.21156 | 0.702144 | -7.76816 |
| Digitoxose | 0.714745 | -0.44649 | -1.06611 | 0.073997 | -0.27614 |
| 1,2,4-Benzenetriol | 0.359327 | -1.11733 | -0.64117 | 1.038924 | -0.63975 |
| L-Dithiothreitol | -0.27137 | -0.13892 | -0.37606 | -0.02504 | -0.18861 |
| D-erythronolactone | -1.77317 | 1.084697 | -0.42764 | -0.99653 | 1.112641 |
| Glutamic acid | 0.458075 | -0.57948 | -0.98704 | 0.106965 | 0.001479 |
| Phenylalanine | 1.045497 | -0.37632 | -1.79009 | 0.444053 | -0.32315 |
| Toluenesulfonic acid | 1.936427 | 0.251222 | -3.23051 | 1.885351 | -1.84249 |
| Tartaric acid | -0.15001 | 6.244619 | 8.420774 | -0.67069 | -14.8447 |
| Fluorene | -1.55738 | 5.540342 | 7.226744 | -10.2046 | -2.00507 |
| Allose | -1.99826 | 0.704863 | -0.00143 | 0.783973 | -0.48915 |
| Lyxose | 9.487671 | -6.15531 | -10.0562 | 3.808595 | 1.915293 |
| Xylose | 2.795525 | -5.40076 | -5.55291 | 1.154094 | 6.004046 |
| 2-ketoadipate | -1.44353 | -2.13319 | -0.08188 | 2.078857 | 0.579749 |
| Ribose | -3.28127 | 1.717227 | 4.51552 | -0.73539 | -3.21608 |
| Xylitol | 0.66364 | -2.4643 | -2.1832 | 1.806382 | 1.177478 |
| Ribonic acid, gamma-lactone | 3.17929 | -2.41758 | -0.76176 | 0.608638 | -1.60858 |
| Levoglucosan | 19.56959 | -11.6568 | -17.2649 | 8.950252 | -0.59815 |
| 6-deoxy-D-glucose | -0.45865 | 0.532572 | -0.02305 | -0.86463 | -0.18624 |
| Fucose | -3.3968 | 0.738 | -0.1073 | 1.039704 | 0.726389 |
| (2R)-2-amino-3-phosphonopropanoic acid | -0.91785 | -0.36585 | 0.204935 | -0.4949 | 0.573668 |
| Flavin adenine | -1.0401 | -0.4037 | -0.26469 | 1.359568 | -0.65107 |
| Putrescine | 0.80053 | -2.23925 | 3.097271 | 2.91259 | -5.57115 |
| Aconitic Acid | 3.916613 | 6.298512 | 4.144413 | 3.965786 | -19.3253 |
| Glucose-1-phosphate | -11.8474 | 10.74467 | 22.07199 | -21.0584 | -0.91083 |
| 3,6-Anhydro-D-galactose | -0.42671 | -0.60543 | 0.494853 | 0.64553 | -1.10824 |
| Glutamine | -1.5015 | 1.593697 | 0.721283 | 0.282788 | -2.09627 |
| 2-Deoxy-D-galactose | -0.86157 | -1.12782 | -0.28149 | 1.578003 | -0.30713 |
| Isocitric acid | -4.47999 | -4.30686 | 12.3443 | 1.971119 | -6.52857 |
| Citric acid | 1.212268 | -0.63057 | -2.98173 | 0.446871 | 0.953161 |
| Shikimic acid | -3.98195 | -76.0316 | -49.1385 | -31.2329 | 159.385 |
| 1,5-Anhydroglucitol | -1.53514 | -4.33594 | 0.805901 | 0.114575 | 3.950602 |
| Dehydroascorbic Acid | -2.86983 | -13.7828 | 0.784522 | 20.36436 | -5.49625 |
| Quinic acid | -1.05733 | 1.784777 | -3.70303 | 1.784777 | 0.190809 |
| Glucose | 1 | -2.98703 | -1.01297 | 1 | 1 |
| Dl-p-Hydroxyphenyllactic acid | 0.099967 | 0.408398 | -0.02064 | -1.21857 | -0.26916 |
| D-Talose | 0.423895 | -5.09382 | -3.85605 | 1 | 6.525974 |
| Lysine | 0.583998 | -0.71571 | -1.32099 | 0.481754 | -0.02905 |
| Sedoheptulose | 31.185 | 22.4726 | 11.20921 | -53.2541 | -12.6127 |
| Tyrosine | 7.503892 | -2.8427 | -6.47991 | 2.651597 | -1.83288 |
| Conduritol b epoxide | 16.72566 | -7.09107 | -7.09107 | -1.77176 | -1.77176 |
| Gallic acid | 0.735655 | 0.760403 | 1.674212 | -3.01972 | -1.15055 |
| 1-Hexadecanol | 1.799968 | -0.35777 | -1.51134 | -1.03708 | 0.106214 |
| Galactonic acid | -2.62826 | 1.613099 | 1.840904 | -2.11225 | 0.286499 |
| Gluconic acid | -1.71421 | -1.51042 | 0.71998 | -0.19609 | 1.70073 |
| Mucic acid | -0.01329 | -0.12815 | -0.34255 | -0.53447 | 0.018461 |
| Palmitic acid | 0.162121 | -0.6284 | -0.76917 | 0.283324 | -0.04787 |
| Myo-inositol | -0.11029 | 1 | -0.11029 | 0.473463 | -2.25288 |
| N-Acetyl-beta-D-mannosamine | 2.067278 | -0.95116 | -2.0754 | -1.6866 | 1.645872 |
| D-Glucoheptose | -1.23583 | -0.74497 | -1.66219 | 1.691197 | 0.951792 |
| Isoxanthopterin | 0.041401 | 0.213832 | 0.041401 | -0.54823 | -0.74841 |
| Phytol | -0.36173 | -0.18424 | -0.3352 | 0.897764 | -1.01659 |
| Fructose 2,6-biphosphate | 0.923225 | -0.65106 | 0.71653 | -1.59109 | -0.39761 |
| Linoleic acid | 0.463186 | 0.325138 | 0.10711 | -1.21256 | -0.68287 |
| Oleic acid | -0.61338 | -0.94179 | -0.02029 | -0.59782 | 1.17328 |
| Linolenic acid | -0.27965 | -0.92958 | -0.51994 | 0.178667 | 0.550513 |
| Stearic acid | 0.222368 | -0.71465 | -0.84105 | 0.12887 | 0.204467 |
| Glucoheptonic acid | 0.106439 | -1.78243 | -1.42984 | 1.226667 | 0.879161 |
| Fructose-6-phosphate | 0.355853 | -1.38561 | -1.05902 | 0.563998 | 0.52478 |
| Glucose-6-phosphate | 0.085498 | -1.43568 | -0.87551 | 0.754354 | 0.471343 |
| Purine riboside | 1.206763 | -1.88845 | -1.30445 | 1.053394 | -0.06726 |
| 6-phosphogluconic acid | 0.576456 | -0.94149 | -0.86622 | 0.021455 | 0.209793 |
| D-erythro-sphingosine | 0.193429 | -0.07094 | -0.30692 | -0.64027 | -0.1753 |
| N-Acetyl-5-hydroxytryptamine | -2.20802 | 0.189114 | 1.540814 | 0.032688 | -0.5546 |
| N-Acetyltryptophan | 0.213941 | -0.00657 | -1.22569 | -0.36358 | 0.381891 |
| Uridine | -2.53809 | 1.859983 | -5.32627 | 2.953309 | 2.05107 |
| Neohesperidin | 3.009852 | -4.70415 | -4.50143 | 3.949116 | 1.246619 |
| Salicin | -3.8241 | 0.547012 | -2.47582 | 6.086333 | -1.33343 |
| DL-dihydrosphingosine | 0.235442 | -0.9974 | -1.90191 | 0.658266 | 1.005603 |
| 2-Monopalmitin | -0.26992 | 0.617151 | -3.70218 | 2.310324 | 0.04462 |
| 1-Monopalmitin | 0.255397 | -0.55378 | -1.59756 | 0.12564 | 0.770308 |
| Phytosphingosine | 0.362855 | 0.164826 | 0.470168 | -1.008 | -0.98985 |
| Xanthosine | 0.371318 | 6.237377 | 4.78078 | -8.55894 | -3.83054 |
| Lactulose | 3.798467 | 1.388505 | -3.45574 | -2.60337 | -0.12786 |
| Cellobiose | 0.230611 | -0.75261 | -1.01963 | 0.166079 | 0.375545 |
| Lactose | 0.146856 | -0.67137 | -0.16148 | -0.73421 | 0.420192 |
| Cellobiose | -0.39497 | 0.529364 | 0.633841 | -0.58029 | -1.18794 |
| Lactobionic Acid | 19.74352 | -37.0101 | -35.3406 | 33.52017 | 18.08707 |
| Maltose | -0.90905 | 1.62312 | -0.91173 | -0.34064 | -0.4617 |
| Digalacturonic acid | 4.002074 | -9.90684 | -0.56174 | 4.134603 | 1.331905 |
| Cellobiotol | -0.42118 | -2.5919 | -3.48328 | 2.494534 | 3.001837 |
| 5'-methylthioadenosine | -6.1342 | -15.0574 | 18.59488 | 11.04043 | -9.44371 |
| Sophorose | -2.13499 | -0.01927 | 0.010186 | -1.15674 | 2.300805 |
| Estra-1,3,5(10)-triene-3,6beta,17beta-triol | -0.36623 | -2.1652 | -1.84483 | 1.360647 | 2.015615 |
| Gentiobiose | 0.944335 | -2.28228 | -2.27629 | 2.159799 | 0.454439 |
| Epicatechin | 0.418011 | -0.00636 | -0.34146 | -1.20893 | 0.138738 |
| Maltitol | -1.01448 | -3.11155 | -3.41899 | 3.96296 | 2.582072 |
| Prunin | 1.957477 | -2.21522 | -1.82388 | 0.308337 | 0.773278 |
| (+)-catechin | -0.18259 | -0.30934 | -0.8408 | 0.194095 | 0.138637 |
| Isomaltose | 0.367919 | -1.78623 | -1.62256 | 0.473656 | 1.567211 |
| 2-Methoxyestrone | 1.735711 | -2.47542 | -1.9529 | 0.377248 | 1.315361 |
| Palatinitol | 0.306574 | -1.43541 | -2.00682 | 1.426922 | 0.708742 |
| Galactinol | -0.44187 | -1.11278 | -1.288 | 1.398978 | 0.44367 |
| Chlorogenic Acid | -0.28078 | -0.68517 | -0.85552 | -0.02244 | 0.843911 |
| 21-hydroxypregnenolone | 1.251124 | -1.5105 | -2.58414 | 0.481525 | 1.361991 |
| Tetrahydrocorticosterone | 1.687262 | -2.64118 | -2.30243 | 1.148846 | 1.107501 |
| 7-alpha-Hydroxycholesterol | 0.144356 | -0.31606 | -0.05194 | -0.66689 | -0.10948 |
| 5-Dihydrocortisol | 0.430829 | 0.118603 | -1.11915 | 0.476127 | -0.90641 |
| Alpha-Tocopherol | 4.060167 | -4.4371 | -2.52353 | 0.024977 | 1.875489 |
| Cholesterol | 11.50455 | -23.0258 | -22.6989 | 21.58767 | 11.63251 |
| Raffinose | -1.41658 | -3.86779 | -1.77259 | 3.174367 | 2.882592 |
| Cholestane-3,5,6-triol, (3beta ,5alpha ,6beta)- | 2.346399 | -1.75575 | -1.57714 | 0.302503 | -0.31601 |
