# Supplementary material for: Karrikin Improves Osmotic and Salt Stress Tolerance via the Regulation of the Redox Homeostasis in the Oil Plant Sapium sebiferum
Source: Front Plant Sci. 2020 Mar 24;11:216. doi: 10.3389/fpls.2020.00216 (PMC7105677; doi:10.3389/fpls.2020.00216)
Supplement: Supplementary file 3 [file Data_Sheet_1.docx]

**
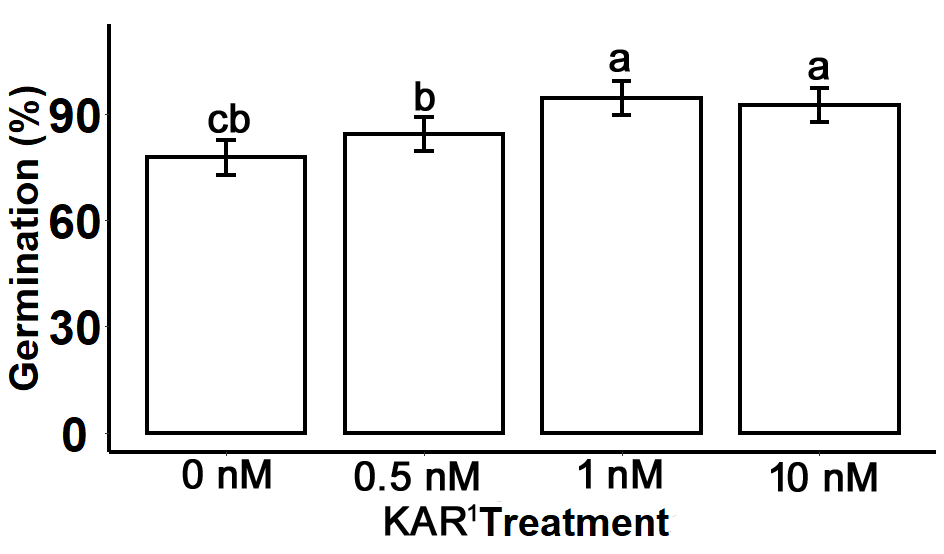
**

**KAR^1^ supplementation in germination medium promoted seed germination in *Sapium sebiferum*.**

For germination investigation, seeds were de-coated with sharp scissors and sterilized by washing twice with 70% ethyl alcohol for 30 seconds, followed by incubation in 20% Sodium hypochlorite for 10 minutes. After that, seeds were washed three times with sterilized water and dried by blotting in sterilized filter papers. We supplemented 0 nM, 0.5 nM, 1 nM, and 10 nM KAR1 in ½ MS medium. We found that 1nM KAR1 supplementation has promoted seed germination significantly, so we used this concentration in all of our experiments. The seed germination test was carried out in five replicates (Petri plates, 12 seeds in each plate). The protrusion of radicle from micropyle was considered as the criterion of seed germination. Germination data were collected 12 days after seed germination and analysed by one-way ANOVA. Multiple comparisons were made by HSD Tuckey’s test at *P<0.05* significant level (n=5).

**List of genes with mRNA sequences**

***SsKAI2***

TTTTAATTCTTCATGCATATTGCAGAATACTCTTATTCATCTATATAGTTGACATTAAAGGCAGACACAAATGATTTTATAGTCACACAATTTTACTCCATTAGCTTGATAGCTTCCATAATCCTAATTAACAATTTATGTAGTTCCATTAAGGAATTAATAACCTAATAAGCACACACAAATTGACCATAGTAAATATAAAATCCAATACTAAATCAAGTGGCAATATCGTGTCGTATGTGTCTAAGAAGAGCCGGAATCCAAATATCTGGCGAGCTCAATTGCGGCAAGTGACCATCCGTCGGTATTATTTCTACAATAGACTCACCACCAAGGTGCTTATGCAAATATTCAGACACAACTACTGGCACAGCTAAGTCCGTAGAACTTTGCAGAATATGACAAGGCACTATCACCAGATGGAGAATTTGTCGCATATCTGTTTGAAATATAGTTTGTGCCACAGATAAGGCTATGTCTGGTCTCATATTAAAGAGAGTGCGGCTGAATTCTTGAACCACCACTGAATCCATATCTCCACCCACCATCAACGGCGCAAACCCTGCGCACCAAGCCTTGTAATTCGATTGCATTGCATCAAATAGTTGGTTTAGATCTTCTTTCTCAAATCCTCCATAGTAATCCACATCGTTCAAATACCTTGGGGAACCAGAGAGCATGATGATTTTGGAGAAAAGATCTGGGCGAGATATGGAGGCAATAGCACCAACCATAGCAGAAACAGAATGACCCACAAAAATACATGATTCAATTTGCAATTCATCCAAAATGGCAAGCAAATCATAAGCAAATCCTTCAAGACTGGCATATCTCTCGAAATCAAAGTAATCTGGGTTAGTAGTACCAGCACCCATATTATCAAACAAGACAACCTTAAAATCATCAACAAGATGAGGGACAAGGTGTTTCCACACAGACTGATCAGTACCAAACCCATGAGCGAGGACCAAGACCTGAGCACCAGTGCCCAAAATCTTGAGGTTATGAGCTTCTTCTACTATACCCATTTCTTGTTTATGGATTTCTCGATTTTGGTCCAAGTTAGTTTCTTGGATTGCGGGGGAATTAGGGGATTTAATAGTATGAAAAAGTGGACTTTCTTTTGGCTGCTAATGTTATTGTGGACGCAAAGGGTGTCGACGGGGGATGATAAGAGATCG

***SsKUF1***

TATACCTATCATGTATCAACGTCCAGTTCAAATAAATAAATAATATTGAAATGTCAACACTAAAGTATTAGGAGAAAAAAAAAATTGATGTAGCTAAGCCCGCACCACATCTTATATAAGCCAATTATTGCCGTGCCGGATTCATCCTCGGCAATATATGAACTGCCACCGCTTCAAATCCCGGTGGCATTTCAACAGTCCGCAGCCTCACCGGCTGTGCCACCACATCCACCACCACAATCTGACCACCGTTGCTGCCACCGCAAACGACGCAAACTCTACCACCACCTACAGCAATCTGCTGTGCTCCTATAAGCCTGTCAGATTGCATTATAGTGTCCCAATCGTCCCTTTTTGGGTCGTACTCCTTAAGTGCACCTTTGCCTTCATCCACCACATACAAAGCCTCCTCATCCATGGCGGCCGCCGGCCCTTTCCACCCATTGAGCATCCCTTCCGGCATATCCTCCCAATCTTCCTTTTTAGCGTCGTAGACCAACCCAACTTTCGCTGCGTCTCCTTTAACATTCACCATACATAGTTTCCCTCTCCATGCTACTGCATCAATGGCCTCCCTGCAAAATTTTCCATCTTTAAGCCCTTTGACCTTCTCCCAGTTGCATTGGCAACCCTTGTTTCTGCTCGTCAAGCAATGATTTTTATGACGAACAAAGTCCCATTTCTCCACTAATTTAGCCACATCCGCCGAGAACTGAGACCCGATTCCACTCGCCGAGTAAATGGCGCCGTTTATTACTCCGGCAGCGCACCAACGGCGGGGAGTGTCGAGGGGAGGCCCAAAACCCCATTTGCTGGAAAGGGGATTGAAAATGAGAGGGCGAGAAAGGGCCGGGGAAAAATTGTCAGTGGTGGCAGCGAGAATAATTAGATGACCGGAAACACAAACAGATTGGATAGGTAGGTGACGGAAAATGAAGGATGGGTGGCGGAGGATAAGGCGGAGAGGAGGGTCTGGTGGCGGAGGAGGAAGAGGGTCCCACCTTGAGGATATAGGATCAAAAGAGAAAAATTGAATTGAATTATCAGTGTTTGTGGAGGATAAAACTATGTATAGAGAAAGAAAAGGACGGAAAGAAGGGGAATAGATTAAACGCCGCCATGAATGGCAAACGGAGTAAAGTAAAGATGGAGGAACAAGAGAGAGGCAAAGCTGAGCTACATGGTTTGGCAAGCCTGGTATAAGATCGGAGTGCTCCTCCTGCAACCGTTGGCGGTGGTCGGGGGTTGATCTTTGGCGTTTGGAGGTGGTCACAACGATGGTTTCCGTGGCCGTAGTCCGGATGGAATTTGCCATATGGCTAGAGAGGAAAGAAAATCAAGAAAAGAGGGAAGAGTACAAATATGGACATGTTCTTGTTTTTTCTTGTTTCTTGTCCTTTTTTTTGTTTTTTTTTTTCTAGTTTTCTGGGATTTAAAGATAAGGACTTGTGGATGAAATAATATTTAAATATTAGTCTATGTCAGATCTTGTAATAAA

***SsDLK2***

GTAAAACATATGCAATTTTGAAATAAATATATTGATTTAAAGCTAGAATAGAAACACTGTCCCTTATCAAATTATGAACACACAAACACTGTCCTAACGGACATTTATTTTTTACCATAATTTCCTGTCCACCAAAAAAAAAAAACTAATATTTATAAGCCAAAGAAATGGAATTCAAGACGAAAGGCCAAGAACTGCATAAAACACATCAAGCAGCTGCTGAGGTGCAGTTAAGTGAGGGAAGTGGCCATTAGTGTCGATAATCTCCACCGTTGATTTCCCTCTGATCTTTTCCTGCATGTAATATGCAACTGAATCGGGAACAACTATGTCGCTAGTTGTCTGAACTATAGTGCAAGGAGTTGTAACCTTGTCAAGAATTTCCCTTTCGTCGCTGTAAAACACAGTTTTTGCCGTAGTTAAGGCTACTTCTGGCCTCATATTAGCCAAACTTTTCGCAAATTTATCTACTGAAGGAGGATCCTTTGCACCCACAGCAAGAGACGCAAAAACTGGAGCCCAGTTGTGGAAATTTGATTCTATGCTCGAGATAATATCGTCGACCTCATATTTCTTAAACCCTCCTTCGTAGTCATCTGTGTTTATGTACCTGGGAGAAGCTCCAATGAGGATGAGCCTCTTAAAAAGCTCAGGTCGTTTAATGGAAGCAATGCAGCCAATCATGCCAGACATAGAGTGGCCAACAAAAACAGAAGATGATTTGAGGCTCAACTCATCTAAGAGACAAATTAAATCATCTGCAAAACCTTGATAGGATGAATACTTATCAGGGTCAAATAGTTGTTGACCTTTAACAGCACCTGAAAAGCTCCAGTCAAAAACAATAACCCTGAAATGTTTAGCCAATTCTGGAATAATATAATCCCAAGCAGATTGATCTCCTCCATATCCATGAGCAAGAACTATGGCCTCGCTACCATCTCCTACGATTCTTGCGTTCATGGCTCTTGAAAGACCATTTTCAAGCATCACCATGCTTGTATATTTTGTGAAGAAACAGTAGGGGAAAAGAAAGCTAGAGAGCAAATTAAAGAGCCAGAGAGAGATACCAGATATGGGTTTGCAGAATAATATGAGGAGA

***SsMAX2***

ATGGCCGCACCAACCGCCGCAACCACCATCCACGACCTCCCCGACGTTATTTTGTCTAATGTTATTGCTTCCATTTCAGATACTCGAACCAGAAACTCAATTTCTCTGGTTAGCCGGAAATTTCTCACTCTTGAAAGAGCTACTCGCACTAACCTCACTCTCCGTGGCAATTCAAGGGACCTTTACATGATTCCTACTTGTTTCAGATCTGTCACTCATCTTGATCTCTCTTTGCTCTCTCCATGGGGTCATTATTTTCTCTCTTCTTCTCACCCCTCTGATCCTCTCCTCCTCGCCCACCGCCTCGCTGTGGCGTTTCCTCTTGTTACTTCTCTCACCCTCTATTGCCGCTCTCCTTCCACTCTCCAGATCCTCCTTCCACGGTGGCCGATGTTGACTTGTATGAAGCTAATTCGGTGGCATCAGCGTCCGTCTTCGTCTCATCTCGGGGCTGATTTTATTCCTTTGCTCGAGCAATGTCAGTTGCTCACTTGTCTCGATCTCTCGAATTTCTATTACTGGACGGAGGATTTGCCTCCTGTTCTTCAAGCTTATCCTGATGTATCGAAATCATTGACTTGCTTGGATCTCCTCACTGTTTCGCTAACTGATGGATTTAAGGCAGATGAGATTCGAACAATCACTGCCGCTTGTTCAAATCTCAGGAAGTTTCTTGTTGCTTGTATGTTCGATCCGAGTTATCTTGGATTTGTAGGAGACGAGACGTTGCTTGCTATTGCTGCGAATTGTCCCAGGTTATCGGTTCTTCATCTGGTAGATACGGCGTCGTTGGGGAGCACGAGGAATGATCCTGAAGACGAAGGGTTAACTGGGGAGGATGCTAGGGTTACAGTTGCTGGATTAGTTGATTTTTTCTCTGGCCTTCCGTTGCTGGAAGAGTTGTTTTTTTGTGTTTGTAAAAGTGTTAGAGATACTTCTGTGGCTCTTGAAGCGCTTAATTCTCGGTGTCCGAAGCTCAAAGTGCTCAAGTTAGGGCAGTTCCACGGCATTTGTATGGCTGTTGAGTCGCAGCTCGATGGGGTTGCTTTGTGTTCGGGGCTGAGATCTTTGTCCATTACCAAGTCCGCGGATTTGACGGATATGGGTTTGATTGAGATTGGTAGAGGATGTTGTAAACTTGCGAAGTTTGAAGTTGAAGGTTGCAAGAAGATTACTATGAAGGGGATGAGGACAATGGCTTGTTTGCTTCACAAGACTCTGGTTGAGGTCAAGATCTCTGCTTGTAAGAATCTCAATGCTGTAGCGTCTTTGCAAGCTTTGGAGCCTATTCGTCACCAGATTGAGTCTCTTCATATCGATTGTATTTGGAATGCTGTGCAACAAGAGGAAAATTATGCTGCTTATCATTGTTTCGATCTCAATGAAGACCTCTTTGAAGGTGCTCAAGATGAGTATTGCAACAAGAGTAAGAGAATTAAGTATTCAGATGAAGCTTGTTTTATGCAAAGCAATGGGGATGGATTTTGGAGCAAGTCCTGGGATAAACTTAGAGTTCTCTCAATCTGGATTGCTGTAGGTGAGCTTCTAACTCCATTGCCAATGGCTGGCCTCCAGGATTGTCCTCGTTTGGAGGAGATTCGGATACAGGTTGAAGGGGATTGCAGGGGCCGGCATAAACCATCACAGCCTGCATTTGGATTGAGCTGCCTTGCACATTATCCTTGTTTGTCAAAGATGCAGTTGAATTGTAGTGACACAATAGGTTTTGCTTTAACTGCCCCCTCTGGACAAATGGATTTGAGCCTGTGGGAGAGGTTTTTCTTGAATGGAATAGGGAATTTGAATCTCAACGAGCTTGATTATTGGCCACCACAAGACCGGGATGTTAACCAGAGGAGTTTATCACTTCCAGGAGCTGGGTTGCTTGCACAATGTCTTTCACTAAGAAAGCTCTTTATACATGGAACAGCACATGAGCATTTCATGATGTTTCTTCTCCGAATTCCTAACCTGAGGGATGTGCAACTTAGGGAAGACTACTATCCCGCACCAGAGAATGATATGAGCACTGAGATGAGAGTGGGATCATGCAGTCGGTTCGAAGATGCCTTGAACGGGCGCCAAATTCTGGACTGA

***SsSMAX1***

ATGAGAGCTGGACTTAGTACGATTCAGCAAACGCTGACCCCAGAGGCGGCGAGTGTTTTAAACCACTCAATTGCGGAAGCGACTCGCCGGAATCATGGTCAGACTACGCCGCTTCATGTTGCGGCGACGCTCTTGGCTTCTCCTTCTGGGTATCTTCGACAAGCTTGTATTAAATCGCACCCAAATTCTTCTCATCCTCTTCAGTGCAGAGCCCTTGAGCTCTGCTTTAGCGTGGCTCTAGAACGGCTACCCACTGCGCAAAATCTTAGCCCAGGTCATGACCCACCAATCTCCAATGCATTAATGGCGGCACTGAAGCGTGCTCAGGCTCACCAGCGGCGGGGTTTCCCTGAACAGCAGCAACAGCCTTTGCTCGCCGTCAAAGTTGAGCTCGAGCAGCTAATTATATCAATTTTAGATGATCCAAGTGTGAGCAGAGTTATGAGAGAAGCTAGTTTTTCAAGCCCAGCTGTTAAGGCCACAATTGAGCAGTCTCTTAACTCTTCATCAAATTCTGCAGCCTCAGCCTCAAATTCAAGCTCATTTGGATTCGGGTTTCGATCTCCTGCAGCAGTACCAGTGCCTACACCAACAACAAATCGGAATTTATATGTGAATCCCAGATTGCAGCAAGGGAATATAGCCCAATCTGGGCAGCATAGGAATGAGGAAGTTAAGCGAGTGGTGGATATTTTGTTGAAGAACAGGAAGAGGAACCCTGTTCTGGTCGGAGAGTTGGAGCCGGAGATGGTGGTAAAGGAGGTCTTGAAAAGAATAGAGAATAAAGAATTAGGAGATGGGTTGTTAAAGAATGTGCAAGTGATTCATTTGGAAAAGGATTTTTTTGATAAAGCTCAAATACCCACAAAGATTATAGAATTAGGGGGTTTAATAGGGAATTTAGATTGCGAAGGAGTAATTCTCGATTTGGGTGATTTAAAATGGCTAGTGGAACAGCCGGTGAGCTTTACTGGTACTGGTGGCGGTGTGCAGCAGCAACAGCAGATTATTTCAGAGGCTGGCCGGGCTGCGGTGACAGAGATGGGGAAACTGTTAGCAAGATTTGGAGAGAAAAGGGTTTGGTTGATAGGTACTGCTACTTGTGAAACATATTTAAGGTGCCAAGTTTATCATCCTTCAATGGAGAATGATTGGGATCTGCAGGCTGTTCCAATTGCTCCAAGAACTCTTGCAGGAAGATTTCCTAGGATTGGAACCAATGGGATTCTTAACAGCTCGGTCGAGTCTTTCTCGCCTCTGAAGGGCTTTCCAACTGTCACACCTAATCTACCAAAGTCAGGTCCTGCTCGAAACACAAGTTGTTGCCCACAGTGTATGCAGAATTATGAGCAAGAGCTTGCCAGAATTGAATCAAAAGATTCCGATAACTCTTCTTCTGAATTTAAATCAGAAGCAACCCATTCGCAGCTGCCGCAGTGGTTGAAAAATGCCAAGTCTCAAGATGGTGATGATAAAACATCGGATAATAAGGAACACAAGAGTCAAGAGTTGCAGAAGAAATGGCATGATGCGTGTAAGCAACTTCATCCTGGCTATCATCAGCCCAATGTTAGCCCTGAGAGACTCACACAAACGGCGCTCTCTACATCAAGTTTGTATAATCCAAACCTGCTTTCTTGCCAACCTTTGATGCCGAAGATAGGTTTGAATAAAAGTCTTGCTGCAACTACACTGTTGAACCCAAATTTGGTGCCCAGCCAATCACCTGCTCGCACTATTACTCCACCAGGAAGCCCTGTGAGAACAGATTTGGTTCTTGGGCGGCCAAAGAGCAAAGACAACACTGCTGAGAAAGTCCACGAAGAGCGAACTAAAAATTTATTGGGTTGTGTTGCTTCTGAGCCACAAATAAAGTTTACTGAATTGCAGGTCAACAAGCTAGATGCTGACTCGTTCAAGAGGCTCCTTAAGGGTCTTATTGACAAGGTGTGGTGGCAGCGAGAAGCAGCATCCGCTGTGGCTACAACTGTGACACAATGCAAAATGGGTAATGGTAAACGGCGGGGAGGTGGATCAAAGGGTGACATTTGGCTATTGTTCACAGGTCCAGACAGGGATGGCAAGAAGAGGATGGCATCAGCTCTTTCAGACCTTGTATGCGGGGCCTGTCCAATAATGGTTTCTCTTGGTTCCCGTCGTGACGATGGGGAACCTACTATGAATTTCCGTGGTAAAACAGCGCTAGATCGTATAGCGGAGGCTGTTAGAAGGAACCCATTTTCAGTAGTCATGCTGGAGGATATCGATGAAGCAGATGTACTAGTTACAGGGAGCATAAAGCGAGCCATGGAAAGAGGTCGGCTTTCTGACTCCCATGGCCGTGAAATCAGTCTCGGGAATGTCATTTTCATCCTTACTGCAAATTGGGTACCAGACAATCTGAAATTCCTAATGAGTGGTGTTTCACTTGATGAGAACAAGCTCGCAAATTTGGCAGGAGGAGGGTGGCAATTGAAACTATCCCTCTATGAGAAAACAGCAAAGCGACGAGCCAATTGGCTGCATGACGAAGAGAGGCCTGCAAAGCCAAGGAAAGACTCAGGATCAGCACTATCATTTGACCTGAACGAAGCAGCTGATGCTGAGGAGGATAAAGCGGATGGCTCTCGCAATTCAAGTGATCTTACAATTGATCATGAAGATGAGAATGTTCTCAATAACAGTCTACTAATGCCAACAACTTCATCAGTACCTCGAGAGCTGCTCAATGCAGTCGATGATGATATAGTTTTCAAACCTATAGATTTTAGCTCTATTCGATCCGAGGTTTCCAACTCTATAACCAAAAGGTTCTGCACCATTATGAGCGAGGAGGGGATCCAGTTGGAAATTAAAGAGGAGGCACTTGAAAAGGTTGCCGGAGGGTTATGGTTAAGCCGAACGAGTTTAGAAGAATTCACTGAGAAAGTAGTGGTTCCAAGCATTCGCCAGCTCAAATTGCAGTTGCCAACATCTGCAGATGAGTCCATGGTTGTGAGGCTCGAATTGGACAGCGATTCAGCTCAACGGAGCCATGGAGATTGGCTGCCGAACAGTGTGAGAGTGGTGGTCGATGGGTTGTGA

***SsWRKY3***

GGCTGAATTTCAGATCCGGAAAGCCAAGCAAAGGAAATCCAAAAGACAGAAAAGAGAAAAAAAACAGTTTCTTGTTTCAGTTGTTTGGATAGGAAGAAAGTAGTAATAGAAACTGAAAAAAGGTTTCTTTTTCTTTGTTATTTTTGCAATGGCTGGAAATGATGCCCCCCACCACCCTCCATCACCATCTTCATCTCCTCCGCCTAAATCTTCCTCTTCTGCCCGCCCTACCACTATCACTTTGCCGCCTCGTTCCTTCAATGAATCCTTCTTCTCCGGTGGACCTCCTATGGGCTTTGGCTTGGGATTAGGATTCAGTCCTGGTCCTATGACTCTCGTCTCTAGCTTCTTCTCCGATTCCGATGACTTTAAATCCTTCTCTCAGCTACTTGCCGGTGCCATGGCCTCTCCTGCAGCTGTGAATAACAAACCTCCCTTTCCTCCTCTTCAGGAGGATAAAACCTCTGCTACTGAAGTTGCTTCTAGGCCAGCTAATTTGTCGATCGCTCCACCTCCGCCGATGTCGACTATGCCTCCGGGATTGAGTCCGGTAGCTCTTCTTGATTCACCGGGGTTTGGACTCTTTTCTCCTCAGGGTCCCTTTGGAATGACTCACCAGCAGGCCCTAGCACAGGTTACTGCCCAGGCTGCACAAGCCAACTCCCAAATGAATATTCAAGCACAGCATTCAGCACCAGCAGCATCTTCAATGCATTTTTCATCTGCTAGTACTAATTCAACTACACAGAAGCAGATGCTACCCTCAATCCCAGACCATAGTTCATCTGTCAAGGAATCTCCTGATGTCTCACATTCTGATCAGAGATCACAAGCTGCTTCACTTGCCGTTGATAAACCCACTGAAGATGGCTACAATTGGCGAAAATATGGGCAGAAACAAGTGAAGGGCAGTGAATACCCTCGAAGTTACTATAAATGCACACATCCCAGTTGTCCTGTCAAGAAAAAGGTTGAGCGATCTCTTGATGGTCAAGTAACTGAAATTATCTATAAAGGACAGCACAACCACCAACCTCCTCCACCTAATAAGCGTGTGAAGGATGCTGGAAGCCTAAATGGAAATTCAAATATTCAGAATAATTTTGAACTAGGTTCCCAACTTCAAAGTGTAAACATGAACAAATTAAAAGATAGGAAGGATCAGGAATCAAGCCAGGCTACTCCTGAACATTTATCTGGTACAAGTGATAGTGAGGAAATTGGTGATACTGAAACTGGTGTAGATGAAAACGATGAAGATGAACCTGATGCCAAGAGAAGGAACACTGAAGTCAAAGCAACTGAACAAGCTTCCTCACATAGGACTGTCACAGAGCCTAGAATCATTGTGCAGACTACTAGTGAAGTTGATCTTTTGGATGATGGTTATAGATGGCGCAAATATGGGCAGAAAGTTGTCAAGGGCAATCCTTATCCTAGGAGTTATTATAAATGCACAACCGCAGGCTGCAAAGTCCGTAAACATGTTGAGCGAGCTGCAGCAGACCCAAGAGCGGTTATAACAACATATGAGGGCAAACATAATCATGATGTACCAGCTGCTAAAGGGAGCAGCCACAGCACAGCAAATACTAAATCATCCGAGCTGAAACAAGAAAATGTAGATAAGCATGCTATAGATAACAGGAGAGACTACGGCACTAATAATCAACAACCCATAGCACGGTTGCGGCTGAAAGAAGAGCAAATAACATAGGTACTTTTGAGTTATCATCAAGAGCTAGAATAACATGGGGATTTAGTATTTACTACGAATCAAGGAATCGCTGCAAGATATCTTTGGTAAAATCTGTTTCAATGCTCTTTGAGGCTATTGTTTTGAGGAAGGCCTATATCACCAGCAGCCATATATATAGGGAATAAATTATAAAAGAAGGTAGACACGTGTAAATCTGGGGCTAGCTTTCATGTATTGAGTTAGAAGGTAATGTTGTATTTAGTTGGTTTAGCAGGCTTATTCTTGTACTTGGCATTATTTTGTACATTATCTGGAAGTAAGTTTGCATATAGTTATTCA

***SsERF5***

ATCCTCATCCTCCTTCTACTTTTCAGAAATCGAGACTCCATTTTCAAAATTCGGAGCAGATTACAAATGGCCACACATACTCAACTAGCTTCTGCCTTGGAGCTCATCAAGCAACATTTATTCGATGATCTTCTTTCTCCTATTCCTTCAACTTCTACTTCCTTTTCTGATTGTAAAATCCACGATGATTTCTTCGAATTTGAAGCTAAGCCTCAAATCATCGATCTCACTTCTCCTAACTCCACTGTTTCAAATCCTCAACTTATCTCTGAATTGAGTGACGATTTCTTCGTTTTCGAATCAAATTCACAAACTATTTCTCAAATCGAATCCAAGCCTCAAGTTTCTTGTTTAAATCGAAAGCCATCACTGAAAATCTCACTGCCTAACAAGACCGAGTGGATCCAATTCACTAACTCCAACCAGCAACCGGTCCAAGCGGATTCAGTCGAAGGCAGAAAGCATTACAGAGGGGTCCGCCAGAGGCCATGGGGCAAGTATGCTGCGGAAATCCGAGACCCGACCCGGAAAGGCACACGTATGTGGCTCGGGACCTTTGATTCTGCTATCGAAGCCGCCAGAGCTTATGATAGAGCGGCGTTTAAGCTGCGAGGCTGTAAAGCCATTCTCAATTTCCCCCTTGAAGCTGGAAAGGAGGGAAATGAACGGAAACGAATCAGAGAAAGTGATGTAGAGAGAGAAGCGAAAAAAGTGGTTAAGAGAGAGGAATCCGACTTATCTGAGTGTGAG

*SsDREB2A*

ATCTGAAGCTCAAAAGCTAAGAACATTTTACTATATCTTTATCTACAAGAAAGAATTTGTTTTAGACAAAACTAGACACCCTCAACCTCCAACAACAATTGCCTGTATAACAACAAGCCAGCCCTCCGTCCTGCCTCAGGGTTGGATTGAAAAGAGAACCAATTTTCCATAGCCAATTTCTCTAATCTAAAAACCTATAGAGAATCGGCTCATTTGTTTGTCAGTAGCAAAAAGAACCAAGTCAGCGGAGCAGAAAATATGTTTGTATGCTGTCCTTATATTTTCAACCTCCTAAAATCCAGTAGGGTACTAATACTATTGAAAAAATACACAAAGCAATACTACTGTCTAGGTCTCTCACACCCATATGATAAATTATGGTTTCATCTTCAGGCAAGAGATATGGCCACATAATCATGTCGCCTCAAACATTGAATCAGACAATCCCAAATTGAAGAATCCTTGATCGTCCAATGGAACATTGTTTTCTTCCTGTCCAACAGGCTTGAAGAAACCAAAGCTGTAATCACCACGCAAAGGCAACTGCTCTGCACGCTGATAACCCCCAATGAATCTATCATCTGGGTTCTGCAGCTGAAAAAATAAATCTAATGGCTGTTCATGCTGCAAATGTCCATTGTCTTGAAATAGGAGCTGTCCACCATCATAATCTGGATTTAGTGGGTGGTTGTCTATGGCCCCAAGCAATTCATCCACGCTGAACATCTCATCCATTGTGATATTTTGCCAGTAATCCTGCCCCTCCAGACCATTTAGAAACCCAAGGTCTTGCATGTTTAGCGACTCATTTTTGCCATCATCTTGCAGATCACAGATGACTTTCTGTTTAAGCTCACAAGTATCATTCTGATTTTCATCTTTCCCTTGCACTTTGTTAAGAAGTATCTTACCCTTGTCACACTTGTCACTCTCTGTAACATCTTCTTGCTCCTTTTTTGCTAGTTGTTTCAGCGCACTACTTGGCACCGAAGCTTCAATTACGGTAGTCTGAGGTTTCATTATTGATTCACCTTCACCATCCCCAACTTTTAAAATATGCTCCTTGGTGTCTTCGACTGCACAAACCTCAGAATGATTTGATGTAGTAGTAAGGTCGGAACCTGCAGGTGTTGCTGCAGAACAATTGCCTGATGGGGTTGCAGCCAAGTGGGAATCCTTTGATGGGCTTGATAATTCAGGGAAGTTCAGCCGGGCATAAGTACCATACATTGCTCGTGCAGCAGCATCATACGCAAGAGCAGCATCATATGCAGTTGGAAATGTGCCAAGCCAGAGTCTAGGCCCTCTATTTGGCTCCCTAATCTCTGCAACCCACTTGCCCCATGTCCGTTGTCTAACACCTCTGTAATTACATCTTGAGTTCTCCGGCCCTCCTTTCCCTTTCATACAACCCTTCCTTGAACCTTTGGCAGGAACTTTACGAGATGATTTATTGTCTTCCTTAGGACACAAATCGAGATAGTTGTTGTATTCTTTCCACTTTTTAAGGGTCTCAGAAACAGTACTACCGCCCCGTCTCTTCCTCTTCCTTGAAGAATCCAAGGGTGTCGAAGTTACATTAGAATGATTGAATGTGCCCATCTCGCTGATTTTATACTGCTCAACCATACCCACCCTTCAATCAACAGGGAGAACTGGGCCAACAGAAATCTGCAAATACTCTCTGAAGCCGTAACCAACAAAGAAACTGCAAAACTCTAGTCTTCGATTCGGATTATCTTCACTACGGATTTTGTTTCTTCCTACTGAAATTTTCGTCTTTTCGCTTTCAAATGAGATTTTAGCTTAATAGCATAGAGAAGTATAGAAGAAATAGCTTCACGAATCAAGAAATCTTTTATGGGAGGTGG

***SsSOS1***

TAACACCAACAAGCTTCTTCCACTAAATTGGAACTGAAAGCCTCATCGAGCAATATTATTATTATTCAGCATGCCTTATTACATAAAGATTTTCAACATCGGGACACCATATAAGTACAAATATGACACCTAACAAAACAGTTTTACGAAGCCTGGCGGAAAGACAGCCTGCTTGGTGAGTCAATTCTCACTATGTGCTCATCCTCAGCACCCGATTCATCACTTGAATTATCTAAAATATTATTGTCATTTGTGTCTGCATTCTGTGGCCGGGAAGCAGAAACTCTCCATGTCGACTGTCTCACCTGTAGGTTCTTCCTTAGAGTAGTAGCTCCTTCTGATCCAACAGAAACCAGCCCACGGCCCTGAAATGATGCAGCTCTTGAAAATGGCATGCTATGTGATCGCTTTACTTGTCTGCTAGAAGAACCGAGGGTATGCCTTTGGACATCAACCATGCTGCCAAAGATGCTCAGCTGCATTGCTCTTGCAGACAAGCTATTTGCTTGTCTGTTTTCAAAATTCTGCTCATGTGACTTTTCCCTGTAGAAATTTTCAGGCCAACTCATCAGAGCACGCTCTCTGCTTGGAAGTCGATGAGGATGATCAACTGTATGTGGCACCAAAGAGGACGACCTTCTCTGAAGAGCAGTATCAGCTTCAAATGCTGCAATGTCAAAAATTATCACTCTTGCTCTTGTCTCGACTTGATACCAGTATCTTTGACAAGAATTACTGGCCGCCCTAACACCTGTTACTTCTTCATTTTGAATGCTTTGGTTTCCATGAGCATATCCAAAGCTCTGATTTTTATGTGGAGGCAACAGTGCTGCAGGTGATGTAATCAATTCTTCCTGAAAGCCGTGGATTTTTATGAATCCTTCTAATAGAAAGCCAATGGAATGATGGGGTACTTCTATTGTTTCTCCCCTTATATGCGTGGTCATCATAGACCTTTCCGCAACAAGAGCTCTCAAATCTTGCATTGCCATTTTTTCAAATATTTGAGGAAGCAAAAGTTTGGCAAGTATTATAGCACTTTCCTGCCACAAGAAGTCTTCCACTGCAGGGTCTGACCGCAGTGCTGAAAGTATTTTCTCACTTTCAATAAAAAAGCAGAGTACTATAGAATCTGTTATCATGTCAGAGATATAAGGCTTTCCAACTAGCACTTCATATATGCCCAATGTGCTCCCATGTGTAAAAGTTGGATTCAAAGAGTGCTTGTTCCTGATGCTGTTACTCTTCCACCTAACAACACCATTAGAAATAAGCCAAACTCCATTTGGCTTGGAGCCCTCTTTGTAAAGTGGCACACCACGTGATTTCATTGTTCCCTTGTTAGAACCTTCTAGATGTTCACGTACTGTAGGGGGAAGAGCCCCCAACATAGGATGCATGCTAATCAAGTCAGTTATTTTGGGAATCTTCACTAAAGGAGGATTCCTCAGAAGCCTTTTTAAGTCAGTCTGTACAGCATCATGAAGATGAAGCATCTCCTTTTCTTCCAAGAGCCCAACCATCTCAAGATTTTGGACATAATCACTTAAATGGCTCAGCACTGAATAAGTTACTTGTCTGGTTTTCACAACTCGCAGAACCTCAGGAAATGAAACACGAACTTCTTCCAAGAATTCTCTGGCTTCTTCTCCTTCTGCCTCACTTTCATTAATGACTTTAGAAGCAATTTCACTATCACCTATAAAGTCATGCAGTTGTCGCCGTGCAATTCTGTGGGCCCGTAGAAATGCAGCACAAATATAGCATGCAGATTCCAATCTCCCTACAATGAAATAAGTAACCAGTTTTCGGGGGCAAATACTTGCTTGAAGAAACCTGTAATAACTTGGGAAATGGAGATTAGCTTTTAAGCCCTTCCAATCACATAGAGGCCCATGAGAAGCCAAGTCAATTGCTTCGTCTACTGATTGCATCAAAATATTTGCAGTAGTTTGTGTTATCCTTCCCTCATTAAGCATTTCCCAGTAAGCAGATTGAATACCATTTAGAAGGCGTATACGGATATCCTTCAAATTTGTTGGGTCTAGATTATTCTCAGATTCAGTTGCATTATGTGGGTGTACACGTCCTTCATCCAAATTATTTAAGCTTGTAATATATCTTCTGACAGTAGGCCAGTCAGCAGGTCCAAGCTCCTCGTCATCTCCAAGATCACCAAAAGCAGCTAATGCTCTGTTCACCATTTCATACTTTGTGTAGTCCAGTATACGTTTCTTGGCAGCCGATAACTTATCCATATCTAGAAAATGCAAAATAAATTGTGTTGTGGATCCATTCACGATAAGTGTCAGAAATACGATTCCACCAGTGAAGAAAACAAACAAAGTTCCTGTTTCGGAAGTGAGATATGCTGAGCTGTCGCTAGTGCGCTTAACAGATAGTGAGAGTGACAATGCAACAGCACCGCGCAAACCTGACCATATGAGAATAGCAGCTTCCTTCCAGTCCAAACCATAACCAAAATATCGCAATAATGGATATAATGCTCCAACAACTACAAACCGAGATAGTTGCACAAAAACATATAGGAGAAAGAGGTATCCCCAAGAATTTCCATGGTTATGAAAAATATCGTGATTGCTGAGGACACTTTCAGCTATAACAACACCACTCAAGATGAAAATTAAGGTATTTGCAATATATGAAACCATCTCCCAAAAGTGATGCAAACTCTGTTGACCATCGCCCTTAAAAGCAGTCCTGGCAGCTGCAGCATAAAACATTCCTAAAGTCATCACTGCCAAAACACCAGAAACATCAGCACCCTCTTGTGCAGCAAAATAAGTGATATAGCTCACGGCAGCTGTCAGTGCAATCTCTATCACCGTGTCATTGAAAATAAAGCCAAGCCACAAAACAGATGCAATCCCAAAAGCAAGACCAATGCCTACAGCCCCAACTGAAACTTTGGCCAGAAATTTGACAATCATTCCTGCATTAGAGCTCTCTCCAAGGACCATCCGATAGAACAACTGATAGACCACAATTGCTGTCCCATCATTCATCAGGGATTCTCCTTCAATTATGGTGCTCAATTTTTTACTTGCACCCAGCTCTTTCAACAGAGCAACAACAGCTACAGGATCAGTAGCACTCAAAAGTCCCCCTAGCAACAGTGAAACTTTCCAGCTCCAGTTATATGGAAAAGTAAGCTTTAGAGCAGATCCAAGACAGAATGTAGAGATCAGAACTCCTGGACCAGCAAGCAGAAGCATTTGTGCCATACATCGCTTGATCTGGTGCATTTCCATTGAAAAAGAACTCTCAAAAAGAAGGGCAGGAAGGAAAACAGCCAATAACAGGTCAGGATCAATATTTGCCCAAATGCGGATGCTGTCCCCAAACTTTCCCAATTGATGATCTGTACCATATTCTATGGATCCAAGAGCAATGCCGATGACGAGTAAAGCAACAGTATAAGGGACTCTAGTTCCACTCAAGAGATGCCTGCAGGCAATCCCAAGCACCAAACTTAACCCGAAGAAAAGGACGGCGTCCGCTGGCTTAGAATCAGAGGCAGAAGAACTCGCCTGAGCCAAAATTCTATACGGGAAATCGCCCTCGATTACAGTCGCCATTTGGATTTCCACTGCAACAGTATCTCCTCACAGAGGAAAAACAAAAACTGAGGAAGTAGCTGAGTTTGGATCGATTGAGTGGTACTTTGGGTTCTGCGTGGCGTAGCGTTATAGTCAAATATAAGAGAGACACGAATA

***SsNCED3***

ATGTCTGAAGATGATTTGCCTTACCATGTTCGTGTTCTTCCTAATGGAGATCTCAAAACAGTGGGTCGATTTGATTTCAATGGACAATTAAAGAGTACGATGATTGCTCATCCTAAGGTCGACCCAATTACGGGGGAACTCTTTGCTCTGAGTTATGATGTTGTCCAAAAACCATATCTCAAGTATTTCAGATTCTCCCCAGATGGTAACAAGTCACCTGATGTTGAAATCCCACTTGATCAGCCCACTATGATGCATGATTTCGCCATAACTGAGAAATTCGTTGTGATTCCTGATCAACAAGTGGTTTTCAAATTGCCTGAGATGATTCGAGGTGGGTCTCCGGTAATCTATGATAAGAAGAAGATGTCGAGATTTGGGATTTTGGACAAGAAAGCTAATGATGCTTCCAAGATTATGTGGGTTGAAGCACCTGATTGTTTCTGTTTTCATCTATGGAACGCATGGGAAGAGCCAGAGACTGATGAAGTGGTGGTGATTGGATCATGTATGACTCCTCCTGATTCCATTTTTAATGAGAGCGAAGAGAAATTAAAAAGTGTTCTATCAGAAATCAGGCTAAATTTGAAGACGGGTAAATCGACTCGACGGGCTATCATCTCTAATCCTGAAGAGCAAGTGAATTTAGAAGCAGGAATGGTGAACAGAAACCTGCTGGGAAGAAAGACTCGACTTGCTTACTTAGCACTGGCAGAGCCATGGCCTAAAGTATCAGGATTTGCCAAAGTAGACCTCTCAACTGGAGAGATACACAAGTATATCTATGGAGAAAGTAAATATGGTGGGGAGCCTCTGTTTATTCCAAGTGGAAGAGGAGATGAAGATGAAGATTGTGGGTATATTTTATGCTTTGTGCATGACGAAAAAGAGTGGAAATCAGAGCTACAGATTGTGAATGCCCAGAATTTGAAGCTAGAAGCAACTGTTAAGCTTCCTTCTCGAGTTCCTTATGGGTTTCATGGCACTTTTATCAGTGAAAATGAATTAAAGAAGCAGGCATAG

***SsNCED9***

CAACAATGAACCAAAGAAAAAAAAAACAGAGTCACGCTTTCTAACTGAGAAGGAAACCCCGGGGACATATCTGCAAGCATCCCTCTGGCAAATCTCCTCTCTAATTTCTCCGACATATATATATATATAATTCTACTATGCCTGCTTCTTTAATTCATTTTCACTGATAAAAGTGCCATGAAACCCATAAGGAACTCGAGAAGGAAGCTTAACAGTTGCTTCTAGCTTCAAATTCTGGGCATTCACAATCTGTAGCTCTGATTTCCACTCTTTTTCGTCATGCACAAAGCATAAAATATACCCACAATCTTCATCTTCATCTCCTCTTCCACTTGGAATAAACAGAGGCTCCCCACCATATTTACTTTCTCCATAGATATACTTGTGTATCTCTCCAGTTGAGAGGTCTACTTTGGCAAATCCTGATACTTTAGGCCATGGCTCTGCCAGTGCTAAGTAAGCAAGTCGAGTCTTTCTTCCCAGCAGGTTTCTGTTCACCATTCCTGCTTCTAAATTCACTTGCTCTTCAGGATTAGAGATGATAGCCCGTCGAGTCGATTTACCCGTCTTCAAATTTAGCCTGATTTCTGATAGAACACTTTTTAATTTCTCTTCGCTCTCATTAAAAATGGAATCAGGAGGAGTCATACATGATCCAATCACCACCACTTCATCAGTCTCTGGCTCTTCCCATGCGTTCCATAGATGAAAACAGAAACAATCAGGTGCTTCAACCCACATAATCTTGGAAGCATCATTAGCTTTCTTGTCCAAAATCCCAAATCTCGACATCTTCTTCTTATCATAGATTACCGGAGACCCACCTCGAATCATCTCAGGCAATTTGAAAACCACTTGTTGATCAGGAATCACAACGAATTTCTCAGTTATGGCGAAATCATGCATCATAGTGGGCTGATCAAGTGGGATTTCAACATCAGGTGACTTGTTACCATCTGGGGAGAATCTGAAATACTTGAGATATGGTTTTTGGACAACATCATAACTCAGAGCAAAGAGTTCCCCCGTAATTGGGTCGACCTTAGGATGAGCAATCATCGTACTCTTTAATTGTCCATTGAAATCAAATCGACCCACTGTTTTGAGATCTCCATTAGGAAGAACACGAACATGGTAAGGCAAATCATCTTCAGACATAGCAAGAAGACGACCATCGAAGTAAACCAAACCAGCGTTAGCAACACCGGTTCCATGGCTCGGATCAACCAGACCGAAAGCCCCACGAGCGTAAAATAGCAAAAGCCGAGCTATACCAGAGTGGCCATGGAGTTCACCTATGGCCTTAGGGAAAATTGAACGTCCTAATTCCCATTCCTGAACCAACCGGTTTGTCTCCGTAAACCGACAGGCGTAGCTAACCGATCCATTTTCGAACCGGACTGCGTGAACCATACCATCACCATCGAAGAAGTGGTGGCCAGCTACTGGTTCATGAAGTGGGTTTGCTCCGTTTCGAAGATAGACACCACGGATACAATCCGGGATATTTCCGGTGAAGGGTAAGTTATGAACGACGGGATGTTCCGGAACCGGAGCAAAGTTGCCGGCGATTTGGACTCTGGGGTCAGCTGTTTTAGGAAGAGGGTGTTGTTTCTCATGGGAAACTAAGGCATTTTCAACAACATCTAAGGCCAAAGCTGCAGCCTTTTGAAGGAAATTCCATTGTTGCTGATGGGGTGTTAATGGTTTATCAGTAGAGGGAGAAGGAGGAGTGTGTTTTTTAGGATTCTTATTAATGGCGGGAGTGGAAATAGCAGGTGAAGCTGGGTAAGTGGTGGTTCTTTTGGATTGTTTAGGAAAATGAAGAATTGAAGGAGTGTGGAGAGAGCAATTAATGTTGGGTTTTCTACTTGGCCTATCTAAACACAAAGAGGAGGAGCAAACCATTCTCACACCTCCTCCTGCTGATGCTGATGCTGAAACCATGATGGACTAATTTTGACCAAATTTAAAATATATGGAGGAAATCTTTTAAAAGAAGGTGATTGGGCAAATGGGTATTAGCTTTTGTTAGTGATTTTGGTGAAATTGAGAGAGAAAATTGGAGAGGGGAAGAAGGA

***SsSnRK2.6***

GATCTTAATGTCTCAAAACAAAAAGGAAGTTTTTAACATAATTAAACAACTGTTAATTAAAAAAAATACATATTATGACATGACGATAATCATCCCAAGACTAGTCCAAAGATTAAATGACAGAGCAGAGCAAGTAGAGTGACAAATAAGGAAACCAAAGAACATATTTCTGGGATGTAATTCAGAGCAACATGACCAACTGATGCTAGACAAGATTAAGACTTCATCGCTGTCAGAAAGCGTGTATAATACTCTACATATATCATGAGGAAGTGGCAAAGCATATTATAGAAGAGTGGAAGCTATCCGTTAAAGGCCATAAATAAAAAAAAGATTTATGCAGATGGATTTTAGCTGATATCAGTAATTTCTAGCCATTAGGTTTCTCCATTTGCGGGAAATGCCATCCTCGAATTTTCACAGCAATCGACCAGTGTGATCAAAATGCTTGTTCCCGGTAAATGTTCACATTGCATACACTATCTCTCCACTGCTATCCATGTCAAGTTCAGGATCACTCTCTAGGTCCTCCTCCATGTCATCATCAATATCCAGGCTACCAGTTAAATATTGATTGAGATTGCGGGCACCTGCTGCTGGTATGGTGGCTTCAGAAATTATCTGCATGATCTCATCAACACATTGTGCTGGCTGATCAAGCTCGTCAAACTGTTTGTTCATTGTATTTTCATCCATGAAATCAGCTGGAAGATTCTTCAGAAACCAGTCGTGATTCCATATCTCGGGAATGCTTATCCTCTTTGCAGGGTCAGCCACAAATATTCTTGACATCAGGTGCTGGCACTCAGGAGATATGTGAACATAGTCGGGAATTGAGTACTGGACTTTCCAAATTCTATCTATCGTCTTGCGGAAGTTCTTAGGCTCCTCAGGGTCTTCAAAGGGATATGCTCCCACCAACATTACATATAAAGTTACCCCACATGACCATACATCTGCAATCTTGCCGTCATATTCCTTCTTTAGTAAAATTTCAGGTGCAATATATGCAGGAGTTCCAACAGTAGATTTTGGTTGTGAATGGAGCACTGATGACTTAGAATAACCAAAGTCACAAATCTTGAGACGGGGTGCTGGGCTTCCATCCAACAATGTATTCTCCAATTTTAAGTCACGGTGGCATACTTGCATTGCATGACAGTAGCTCACTCCTGATATAAGTTGTTGGAAGAAAAAGCGTGCCTCATCTTCACTGAAGCGGCCTGCATTGCATATCCGCTCAAAAAGCTCCCCACCAGATGCATACTCCATCACAATAGCCAGGTGTGTTGGTGTCAAGATAACCTCTTTGAATCTAACAATGTTAGGATGCCTCAATGACCTGTGGTTAATAATTTCCCTTTGTACATTTTCATCTATCTTCTCACCTCTCTCGATATACTTGACGGCAACAAGCTCGTCGGTCTGCTTGTCTCTCATCAACCTTGCTACCCCGAAGTTCCCAGACCCGATATCTCGCACAAGCTCGTACCGGTCACTATCGTGCATAATCGGCATATCCATACCCGGACCTACTGTGACAGCCGATCGATCCATTTCTATCTATTCTCAATCTCGACTACAAAAACAGAAGAAACACTAGAAACCCAAAAACTCTTCCTCGCAAAAAGAAAAAACCCAGCAAAATAAAAAAGTTCAAGTCAATCTTTCACATTATGATCAACATCAGAGGAACCCCTGATCATGAGAAATCGAAACGAGAAGGAGGTAGTGCTGGAGGTGAAGTGGCGAAACGTGAGCCACTGGACTGGATGGTAAAGAAAGACGTAAAAGATGGGTCTAACCTCGTGAAAACCTCACCCACCCATATAGGTTCTGCTTATTTTTTTTCAGGTAATTCGGTAAGAGAAAGCAAGAAAGAGCTTGCAGAGAAAGAACGAAAGAAAGAAACGACAGATAGCTGTAGCTCGTGAAACCAGCTGCTTTATTCACCTTCTGAAAAAACCCCAAAAAGACTCCAACTTTGGTTAATTTACATGCTTCCTCTCTCTCAATTAGGAAACCTAATACTTCCCTTTCGTTTCTCTTTTTCTCTGCTTTTGCGTGATGTGTGGGGTTTTATCCGAAACTGTTGGTGAGAGAGAGAGAG

***SsSnRK 2.3***

AAGCAATTCCCTTTCACTTCAGCTTTTTCTCATTCGTGGCCATGAAACCCATACGCAGATCCTCATTTCTCAACAGCAACAAGTAGAGGGACTGTCCTAATTCAAGTGAAGCAACATATGCATCATTCCATCTTCTTTCAGCTTTATTATATGTCTTATATAGGGATTTAAGTCAAATATTTTTCTAAATATAAATTCTTACCTTTTCTTCCAATCATATATAATCATTATTCTTGTCAATGGAGAGATACGAAATACTGAGAGATATTGGGTCAGGGAATTTTGGAGTTGCTAAGCTTGTTAGAGATAAATGGAGTGGTGAACTCTTTGCTGTCAAGTATATTGAGAGAGGCCAAAAGATTGATGAGCATGTGCAGAGAGAGATCATGAACCACAGGTCATTGAAGCATCCAAATATAATTAGATTCAAAGAGGTATTTCTGACACCAACCCATTTAGCCATAGTAATGGAATATGCAGCAGGGGGAGAACTTTTTGAGAGAATATGCAATGCTGGTAGATTCAGTGAAGATGAGGCAAGATATTTCTTTCAACAACTCATATCAGGAGTTAGCTACTGCCATTCCATGCAAATTTGCCATAGAGATCTTAAGCTGGAAAATACCCTCTTAGATGGAAGCTCAGCACCTCTTCTTAAGATATGTGATTTTGGGTATTCAAAGTCTTCTGTATTGCATTCTCAACCCAAGTCTACAGTGGGAACACCAGCCTATATTGCACCAGAGGTCTTATCAAGAAAAGAATATGATGGGAAGATTGCAGATGTTTGGTCTTGTGGGGTTACCTTGTATGTGATGCTGGTAGGGGCTTATCCATTTGAAGATCCTGAAGACCCAAGAAACTTCAGGAAAACTATTCAGAGGATTCTTAGTGTCCACTACTCAATTCCAGACTATGTTAGAGTTTCAACAGAATGTAAACATCTTCTATCTAGGATTTTTGTAGCTAATCCTGAAAAGAGGATAACAATACCAGAAATTGAGAAGCATCCTTGGTTCCTGAAGAGCTTGCCTGAAGAATTCAAGGAAGTGGAGGAAGATGGCTTGCAAAATGATGAAAAGGATGAAGAATCTCAAAGCATTGAGGAAATAATAGGTATAATTCAGGAGGCAAGAAAACCTGCAGATGAGCCTAAGATTGGAGGAAATTTGTTTGGGGGGAGCATGGATCTTGATGATACTGACATTGATGATATAGAGACAAGTGGTGATTTTGTTTGTGCATTGTGATTGCAACTTTAAGAATTTAAATCATGGGAATTTTCTATTCAATCTTAAATGAAATGGCAGTTTCAGATTAGGACCATATTTAATTTCCATGCATAGAGTGCTAATGTTAAATTTTCATTTGTATAAAAGTTTGTGTTTATGGGATAATATAGATTATGTATGCTACTTTTAATTATATTCCTCCATCTTGATCTGTACTAATTTTATAAAGAGGATATATTTCAG

**SsUBQ10**

ATGCAGATCTTTGTTAAGACTCTCACCGGAAAGACAATCACCCTCGAGGTGGAAAGCTCCGACACCATCGACAACGTTAAGGCCAAGATCCAGGATAAGGAGGGCATTCCTCCGGATCAGCAGAGGCTTATTTTCGCCGGCAAGCAGCTAGAGGATGGCCGTACGTTGGCTGATTACAATATCCAGAAGGAATCCACCCTCCACTTGGTCCTCAGGCTCCGTGGTGGTATGCAGATTTTCGTTAAAACCCTAACGGGAAAGACGATTACTCTTGAGGTGGAGAGTTCTGACACCATCGACAACGTCAAGGCCAAGATCCAAGACAAAGAGGGTATTCCTCCGGACCAGCAGAGGCTGATCTTCGCCGGAAAGCAGTTGGAGGATGGCAGAACTCTTGCTGACTACAATATCCAGAAGGAGTCCACCCTTCATCTTGTTCTCAGGCTCCGTGGTGGTATGCAGATTTTCGTTAAGACGTTGACTGGGAAAACTATCACTTTGGAGGTGGAGAGTTCTGACACCATTGATAACGTGAAAGCCAAGATCCAAGACAAAGAGGGTATTCCTCCGGACCAGCAGAGATTGATCTTCGCCGGAAAACAACTTGAAGATGGCAGAACTTTGGCCGACTACAACATTCAGAAGGAGTCCACACTCCACTTGGTCTTGCGTCTGCGTGGAGGTATGCAGATCTTCGTGAAGACTCTCACCGGAAAGACCATCACTTTGGAGGTGGAGAGTTCTGACACCATTGATAACGTGAAAGCCAAGATCCAGGACAAAGAGGGTATCCCACCGGACCAGCAGAGATTGATCTTCGCCGGAAAGCAACTTGAAGATGGAAGAACTTTGGCTGACTACAACATTCAGAAGGAGTCCACACTTCACTTGGTCTTGCGTCTGCGTGGAGGTATGCAGATCTTCGTGAAGACTCTCACCGGAAAGACTATCACTTTGGAGGTAGAGAGCTCTGACACCATTGACAACGTGAAGGCCAAGATCCAGGATAAGGAAGGAATCCCTCCGGACCAGCAGAGGTTGATCTTTGCCGGAAAACAATTGGAGGATGGTCGTACTTTGGCGGATTACAACATCCAGAAGGAGTCGACCCTTCACTTGGTGTTGCGTCTGCGTGGAGGTATGCAGATCTTCGTCAAGACTTTGACCGGAAAGACCATCACCCTTGAAGTGGAAAGCTCCGACACCATTGACAACGTCAAGGCCAAGATCCAGGACAAGGAAGGTATTCCTCCGGACCAGCAGCGTCTCATCTTCGCTGGAAAGCAGCTTGAGGATGGACGTACTTTGGCCGACTACAACATCCAGAAGGAGTCTACTCTTCACTTGGTCCTGCGTCTTCGTGGTGGTTTCTAA
